# Daughterless, the *Drosophila* orthologue of TCF4, is required for associative learning and maintenance of the synaptic proteome

**DOI:** 10.1242/dmm.042747

**Published:** 2020-07-30

**Authors:** Laura Tamberg, Mariliis Jaago, Kristi Säälik, Alex Sirp, Jürgen Tuvikene, Anastassia Shubina, Carl Sander Kiir, Kaja Nurm, Mari Sepp, Tõnis Timmusk, Mari Palgi

**Affiliations:** 1 Department of Chemistry and Biotechnology, Tallinn University of Technology, Akadeemia tee 15, Tallinn 12618, Estonia; 2 Protobios LLC, Mäealuse 4, Tallinn 12618, Estonia

**Keywords:** TCF4, Daughterless, Pitt–Hopkins syndrome, Intellectual disability, *Drosophila melanogaster*, Appetitive associative learning, Negative geotaxis

## Abstract

Mammalian transcription factor 4 (TCF4) has been linked to schizophrenia and intellectual disabilities, such as Pitt–Hopkins syndrome (PTHS). Here, we show that similarly to mammalian TCF4, fruit fly orthologue Daughterless (Da) is expressed widely in the *Drosophila* brain. Furthermore, silencing of *da*, using several central nervous system-specific Gal4 driver lines, impairs appetitive associative learning of the larvae and leads to decreased levels of the synaptic proteins Synapsin (Syn) and Discs large 1 (Dlg1), suggesting the involvement of Da in memory formation. Here, we demonstrate that *Syn* and *dlg1* are direct target genes of Da in adult *Drosophila* heads, as Da binds to the regulatory regions of these genes and the modulation of Da levels alter the levels of *Syn* and *dlg1* mRNA. Silencing of *da* also affects negative geotaxis of the adult flies, suggesting the impairment of locomotor function. Overall, our findings suggest that Da regulates *Drosophila* larval memory and adult negative geotaxis, possibly via its synaptic target genes *Syn* and *dlg1*. These behavioural phenotypes can be further used as a PTHS model to screen for therapeutics.

This article has an associated First Person interview with the first author of the paper.

## Abstract (precis)

**Summary:** Human TCF4, a bHLH transcription factor, is associated with intellectual disability and schizophrenia. Here, we propose a *Drosophila* model for human disease studies using the fruit fly TCF4 orthologue, Daughterless.

## INTRODUCTION

Transcription factor 4 (TCF4, also known as ITF2, E2-2, SEF2, etc.) belongs to the family of class I basic helix-loop-helix (bHLH) transcription factors, also called E-proteins (Murre et al., 1994). E-proteins bind to the DNA Ephrussi box (E-box) sequence CANNTG as homodimers or heterodimers with class II bHLH transcription factors (Cabrera and Alonso, 1991). TCF4 should be distinguished from TCF7L2, a downstream effector of the Wnt signalling pathway that is also referred to as TCF4 (T cell factor 4). TCF4 is essential for a range of neurodevelopmental processes including early spontaneous neuronal activity, cell survival, cell cycle regulation, neuronal migration and differentiation, synaptic plasticity and memory formation (Chen et al., 2016; Crux et al., 2018; Forrest et al., 2013; Hill et al., 2017; Jung et al., 2018; Kennedy et al., 2016; Kepa et al., 2017; Li et al., 2019; Page et al., 2018; Thaxton et al., 2018). Genes involved in pathways including nervous system development, synaptic function and axon development are TCF4 targets (Forrest et al., 2018; Xia et al., 2018). Furthermore, TCF4 regulates the expression of ion channels Na_v_1.8 and K_v_7.1 (Ekins et al., 2019; Rannals et al., 2016). Recent insights into the mechanisms of activation of TCF4 show that TCF4-dependent transcription in primary neurons is induced by neuronal activity via soluble adenylyl cyclase and protein kinase A (PKA) signalling (Sepp et al., 2017). In addition to the nervous system, TCF4 has been shown to function in the immune system during the development of plasmacytoid dendritic cells (Cisse et al., 2008; Grajkowska et al., 2017).

Deficits in TCF4 function are associated with several human diseases. TCF4 haploinsufficiency causes Pitt–Hopkins syndrome (PTHS; OMIM #610954) (Amiel et al., 2007; Brockschmidt et al., 2007; Zweier et al., 2007). As reviewed in an international consensus statement (Zollino et al., 2019), patients with PTHS have severe intellectual disability, developmental delay, intermittent hyperventilation periods followed by apnea, and display distinct craniofacial features. Currently, there is no treatment for PTHS, but dissecting the functional consequences triggered by mutated *TCF4* alleles could reveal attractive avenues for curative therapies for this disorder (reviewed in Rannals and Maher, 2017). Large-scale genome-wide association studies revealed single nucleotide polymorphisms in *TCF4* among the highest risk loci for schizophrenia (SCZ) (Talkowski et al., 2012). Consistently, *TCF4* is involved in SCZ endophenotypes, such as neurocognition and sensorimotor gating (Lennertz et al., 2011a,b; Quednow et al., 2011). Furthermore, many genes that are mutated in SCZ, autism spectrum disorder and intellectual disability patients are TCF4 target genes (Forrest et al., 2018). How deficits in TCF4 function translate into neurodevelopmental impairments, and whether TCF4 plays an essential role in the mature nervous system, is poorly understood.

We have previously demonstrated that TCF4 function can be modelled in *Drosophila melanogaster* using its orthologue and the sole E-protein in the fruit fly, Daughterless (Da) (Tamberg et al., 2015). PTHS-associated mutations introduced to Da lead to similar consequences in the fruit fly as do the same mutations in TCF4 *in vitro* ranging from hypomorphic to dominant negative effects (Sepp et al., 2012; Tamberg et al., 2015). Furthermore, human TCF4 is capable of rescuing the lack of Da in the development of the *Drosophila* embryonic nervous system (Tamberg et al., 2015).

Da is involved in various developmental processes including sex determination, neurogenesis, myogenesis, oogenesis, intestinal stem cell maintenance and the development of the eye, trachea and salivary gland (Bardin et al., 2010; Bhattacharya and Baker, 2011; Brown et al., 1996; Castanon et al., 2001; Caudy et al., 1988; Cline, 1978; Cummings and Cronmiller, 1994; King-Jones et al., 1999; Massari and Murre, 2000; Smith et al., 2002; Wong et al., 2008). In the developing nervous system, the role of Da is well established during neuronal specification as an obligatory heterodimerization partner for proneural class II bHLH transcription factors (Cabrera and Alonso, 1991; Powell et al., 2008). However, the functional role of Da following neurogenesis and nervous system maturation remains unknown.

Here, we set out to characterize the expression of Da in the nervous system. To this end, we created *Drosophila* lines in which Da protein was endogenously tagged with either 3xFLAG or sfGFP epitope tags. We showed that Da is broadly expressed in the larval central nervous system (CNS), including in populations of Kenyon cells contributing to the mushroom body, which is the memory and learning centre of insects. To test whether Da is involved in learning and memory formation in the fruit fly, we used the appetitive associative learning paradigm in larvae (Michels et al., 2017). In this assay, silencing of *da* by several CNS-specific Gal4 drivers resulted in impaired learning and memory formation. Knockdown of *da* using 30Y-Gal4 also impaired negative geotaxis of adult flies. These phenotypes were moderately improved by adding resveratrol or suberoylanilide hydroxamic acid (SAHA) to the food substrate. Therefore, our results show that knockdown of *da* combined with appetitive associative learning paradigm or negative geotaxis assay is further applicable for screening potential therapeutics for the treatment of PTHS, as well as putative genetic interactors of Da and by proxy, TCF4. Furthermore, silencing of *da* resulted in a decreased level of the synaptic proteins Synapsin (Syn) and Discs large 1 (Dlg1) in third instar larval brains. We also demonstrated that Da binds to several areas in the *dlg1* gene and to the *Syn* promoter region in adult *Drosophila* heads, and that overexpression of *da* increases *Syn* and *dlg1* mRNA levels in the adult heads. Collectively, we have shown for the first time that Da is required to sustain elements of the synaptic proteome in a mature nervous system positing a post-developmental function for Da and possibly TCF4.

## RESULTS

### Da is expressed at all developmental stages of the fruit fly

**Fig. 1.**
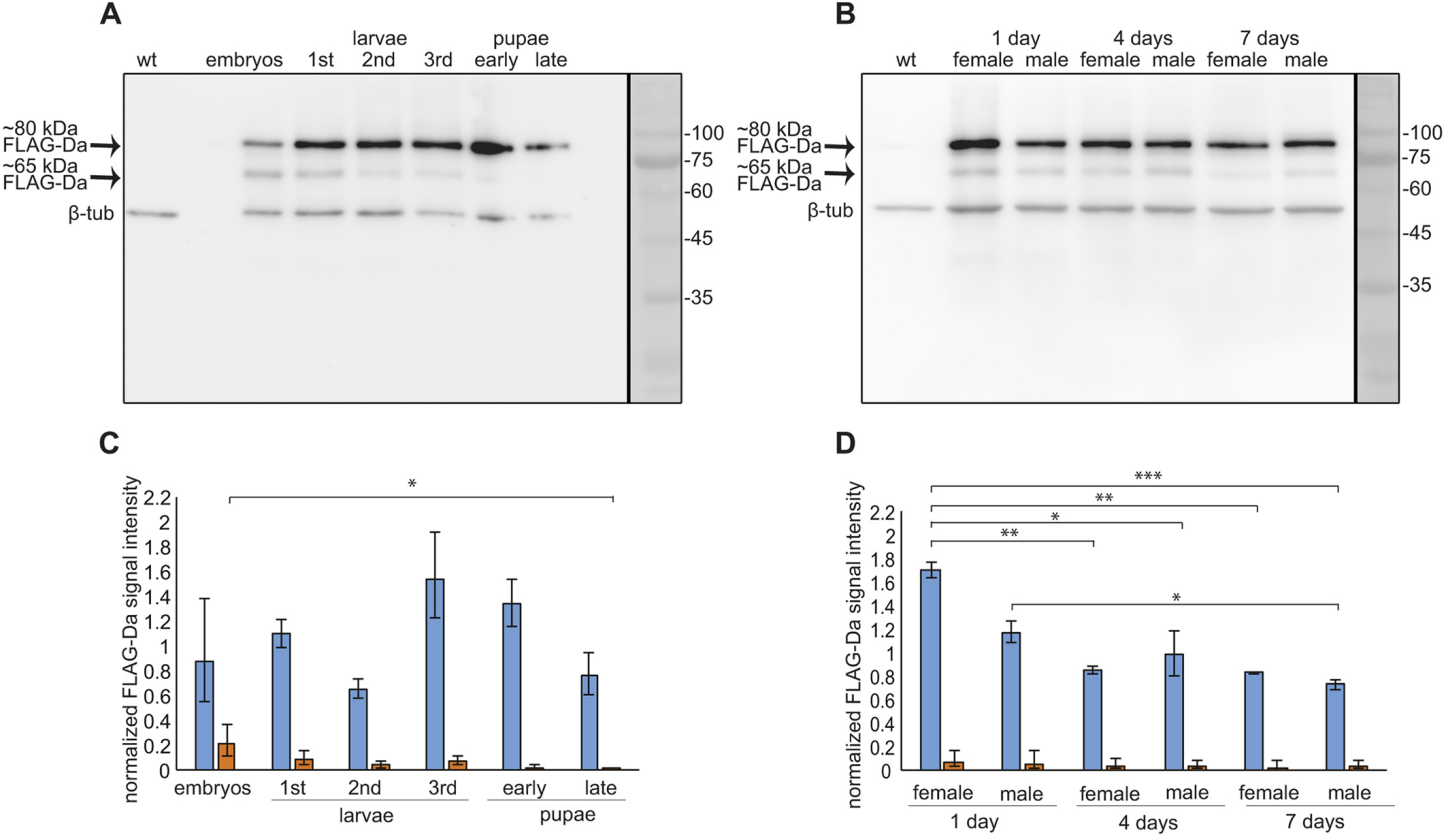
**Da is expressed in all developmental stages of the fruit fly.** (A,B) The 3xFLAG-Da fusion protein is expressed throughout fruit fly development. Western blot analysis carried out using anti-FLAG antibody reveals two FLAG-Da-specific signals, one at ∼80 kDa and the other at ∼65 kDa. The w^1118^ wild type (wt) serves as a negative control. The numbers on the right side indicate the molecular weights of proteins in kDa. (C,D) Results of densitometric analysis of western blot. The 3xFLAG-Da signals were normalized using β-tubulin signals. The mean results from three independent western blots are shown. The blue bars represent the mean intensity of the 80 kDa protein signal and the orange bars represent the mean intensity of the 65 kDa protein signal. Data are mean±s.e.m. Statistical significance is shown with asterisks between the groups connected with lines. *P<0.05, **P<0.01, ***P<0.001, one way ANOVA with post-hoc Bonferroni test.

Although the expression of Da protein has been studied in fruit fly embryos, ovaries, larval optic lobes and imaginal discs using various anti-Da antibodies (Andrade-Zapata and Baonza, 2014; Bhattacharya and Baker, 2011, 2012; Brown et al., 1996; Cronmiller and Cummings, 1993; Li and Baker, 2018; Tanaka-Matakatsu et al., 2014; Yasugi et al., 2014), its expression during adulthood remains largely uncharacterized. Therefore, we first aimed to study Da expression throughout the development of the fruit fly using immunoblot analysis. As there are no commercial antibodies available that recognize Da, we used the CRISPR/Cas9 system to create transgenic flies in which Da was N-terminally tagged with 3xFLAG epitope. The resulting *3xFLAG*-*da* line was maintained in a homozygous state, indicating that the tagged Da protein is functional as both *da* null mutations and *da* ubiquitous overexpression lead to embryonic lethality (Caudy et al., 1988; Giebel et al., 1997). We then characterized Da expression throughout development, and in adult *Drosophila* heads of the *3xFLAG*-*da* line, by performing immunoblot analysis with anti-FLAG antibodies. During development, we compared Da expression from embryonic to late pupal stages (Fig. 1A,C). In adults, we analysed the Da levels from the heads of 1-, 4- and 7-day-old males and females (Fig. 1B,D). In addition to the expected FLAG-Da signal at ∼80 kDa, we identified a previously uncharacterized lower molecular weight (∼65 kDa) Da signal (Fig. 1A,B, Fig S1, Fig. S2A). This signal was also present in a western blot of embryos using anti-Da antibody in flies overexpressing Da under the ubiquitous strong driver *da^G32^-Gal4* but not with nervous system-specific *R123B08-Gal4* (Fig. S1). We found no significant differences in 80 kDa Da protein expression throughout development from the embryonic stage to pupariation (Fig. 1C). During adulthood, Da expression was highest in the heads of 1-day-old females and decreased thereafter in both males and females (Fig. 1D). Expression of ∼65 kDa Da decreased during development, with highest levels at the embryonic stage (Fig. 1C,D). This ∼65 kDa Da protein also seemed to be mostly non-neural as its expression level was very low in larval brains (Fig. S2).

### 3xFLAG-Da retains the transactivational capability of Da in HEK293 cells

**Fig. 2.**
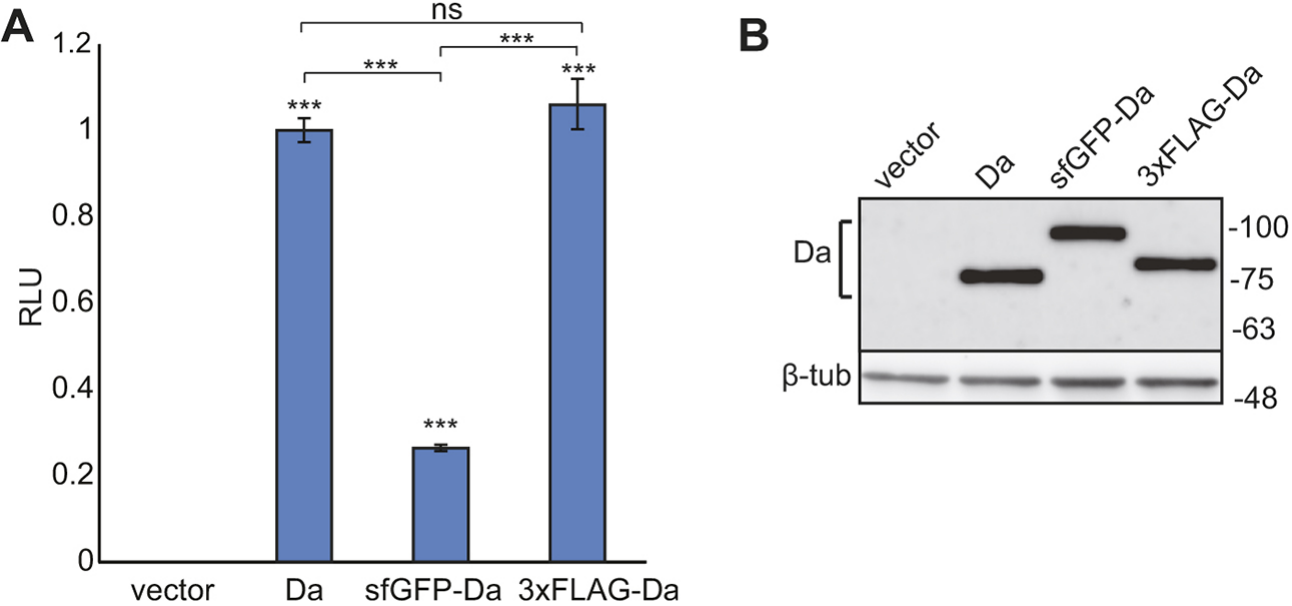
**Transactivational capability of Da is unaffected by an N-terminal 3xFLAG tag, but is reduced by an sfGFP tag.** (A) HEK293 cells were co-transfected with constructs encoding wild-type *da*, tagged *da* or empty vector, a firefly luciferase construct carrying 12 µE5 boxes with a minimal promoter, and a *Renilla* luciferase construct without E-boxes for normalization. Luciferase activities were measured and data are presented as fold-induced levels compared to the signals obtained from cells transfected with the wild-type *da* encoding construct. The mean results from six independent transfection experiments performed in duplicates are shown. Data are mean±s.e.m. Statistical significance compared to cells transfected with empty vector is shown with asterisks above the bars. Statistical significance between the groups is indicated with brackets. ***P<0.001; ns, not significant; one way ANOVA with post-hoc Bonferroni test. RLU, relative luciferase unit. (B) Western blot from transfected HEK293 cells using anti-Da antibody dam109-10. Wild-type Da, sfGFP- and 3xFLAG-tagged Da are all expressed at equal levels. Numbers on the right side indicate the molecular weight of the proteins in kDa.

In addition to *3xFLAG*-*da*, we also created *sfGFP-da* flies, in which Da is tagged with superfolder green fluorescent protein (sfGFP) in the same N-terminal position. This line was also maintained in a homozygous state, indicating that sfGFP tag does not interfere with the function of Da *in vivo.* To determine whether N-terminal tagging of Da proteins influences their transactivation capability, we used an *in vitro* luciferase reporter system in which the expression of the *luciferase* gene was controlled by E-boxes with a minimal promoter. Therefore, we cloned the *3xFLAG*-tagged or *sfGFP*-tagged *da* from the genomes of the tagged lines into mammalian expression vector pcDNA3.1 and overexpressed these constructs in HEK293 cells. The luciferase reporter assay showed that compared to wild-type Da the transactivational capability of 3xFLAG-Da was unchanged (Fig. 2A). In contrast, the transactivational capability of sfGFP-Da was significantly reduced (Fig. 2A). To determine whether the effects seen in the luciferase reporter assay were due to differential expression levels of the Da proteins used, we also performed western blot analysis, which revealed that both *3xFLAG-da* and *sfGFP*-*da* constructs were expressed at equal levels (Fig. 2B). This suggests that the 3xFLAG tag, unlike the sfGFP tag, does not interfere with the expression and transcriptional activity of Da. Correspondingly, we focused on using 3xFLAG-Da flies in subsequent experiments.

### Da is widely expressed in the third instar larval brain

**Fig. 3.**
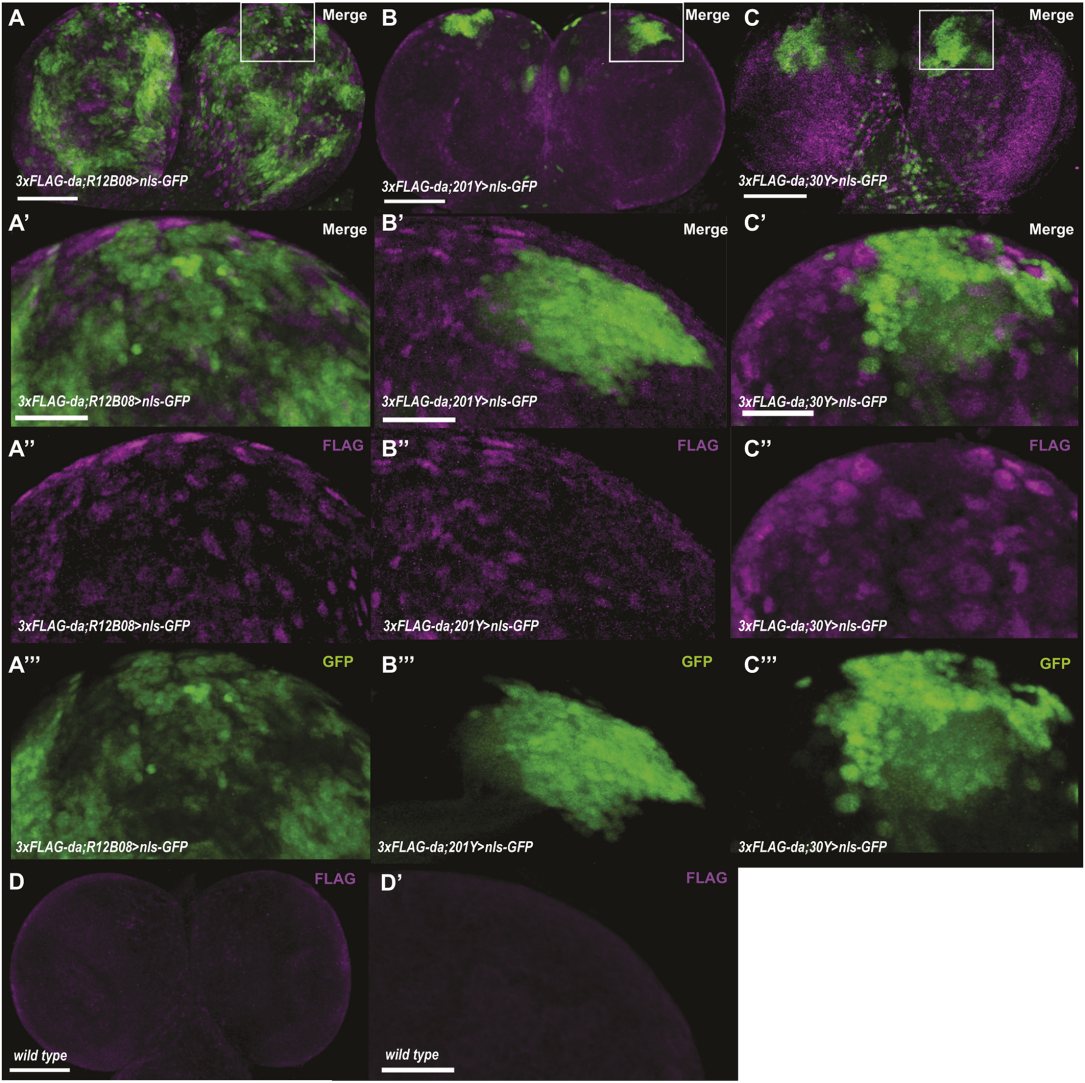
**Da is widely expressed in the third instar larval brain, including several Kenyon cells of the mushroom body.** (A-A‴) *R12B08-Gal4* is expressed widely in the larval brain. (B-C‴) *da* is expressed in some of the mushroom body Kenyon cells in third instar larval brains marked by *201Y-Gal4* (B-B‴) and by *30Y-Gal4* (C-C‴). nls-GFP expression shows driver expression pattern (A‴,B‴,C‴). The expression of 3xFLAG-Da (A″,B″, C″). Wild-type third instar larval brains showing unspecific binding of anti-FLAG antibodies (D,D′). Scale bars: 70 μm (A,B,C,D; boxes show the area represented in A′-C‴); 20 μm (A′,B′,C′,D′).

Next, we used the *3xFLAG-da* line to characterize the expression of Da in the third instar larval brain. Da was expressed weakly and ubiquitously throughout the larval CNS, with stronger expression detected in some nuclei and cytoplasm of specific cells (Fig. 3A″,B″,C″). Mutations in, or deletion of, one of the *TCF4* alleles lead to PTHS in humans. One of the hallmarks of PTHS is severe learning disability, and it has been shown that *TCF4* is highly expressed in the adult human and rodent hippocampus, which is the brain structure involved in learning and memory (Sepp et al., 2011; Jung et al., 2018). Additionally, single-cell RNA sequencing data have shown that *da* mRNA is expressed widely in the adult fly brain and also in mushroom body Kenyon cells (Davie et al., 2018). Therefore, we attempted to determine whether TCF4 homologue Da is expressed in the mushroom body, the brain structure of insects responsible for learning and memory. To facilitate this, we deployed the UAS-Gal4 binary expression system (Brand and Perrimon, 1993) by combining the *3xFLAG*-*da* line with different driver lines with expression in the mushroom body. Resulting lines with *3xFLAG*-*da* and *Gal4* were then combined with nuclear-targeted *UAS-nls-GFP*. The *R12B08-Gal4* line directed *Gal4* expression under the control of the single intron of *da* in most regions of the brain, including the mushroom body (Fig. 3A,A‴). Two mushroom body-specific drivers, *201Y-Gal4* and *30Y-Gal4*, were used to express nuclear GFP to visualize Kenyon cells (Fig. 3B,B‴,C,C‴). We observed that the expression of 3xFLAG-Da and *R12B08>nls-GFP* overlapped in many areas of the third instar larval brain (Fig. 3A′). With *201Y-Gal4* and *30Y-Gal4*, the mushroom body-specific driver lines, 3xFLAG-Da showed partial co-expression in cells contributing to the third instar mushroom body (Fig. 3B′,C′). Thus, Da is expressed broadly in the CNS of third instar larvae, including a portion of the mushroom body. Wild-type larval brains were used to validate the specificity of anti-FLAG antibodies (Fig. 3D,D′).

### Silencing of *da* in the CNS leads to impaired memory of the larvae

**Fig. 4.**
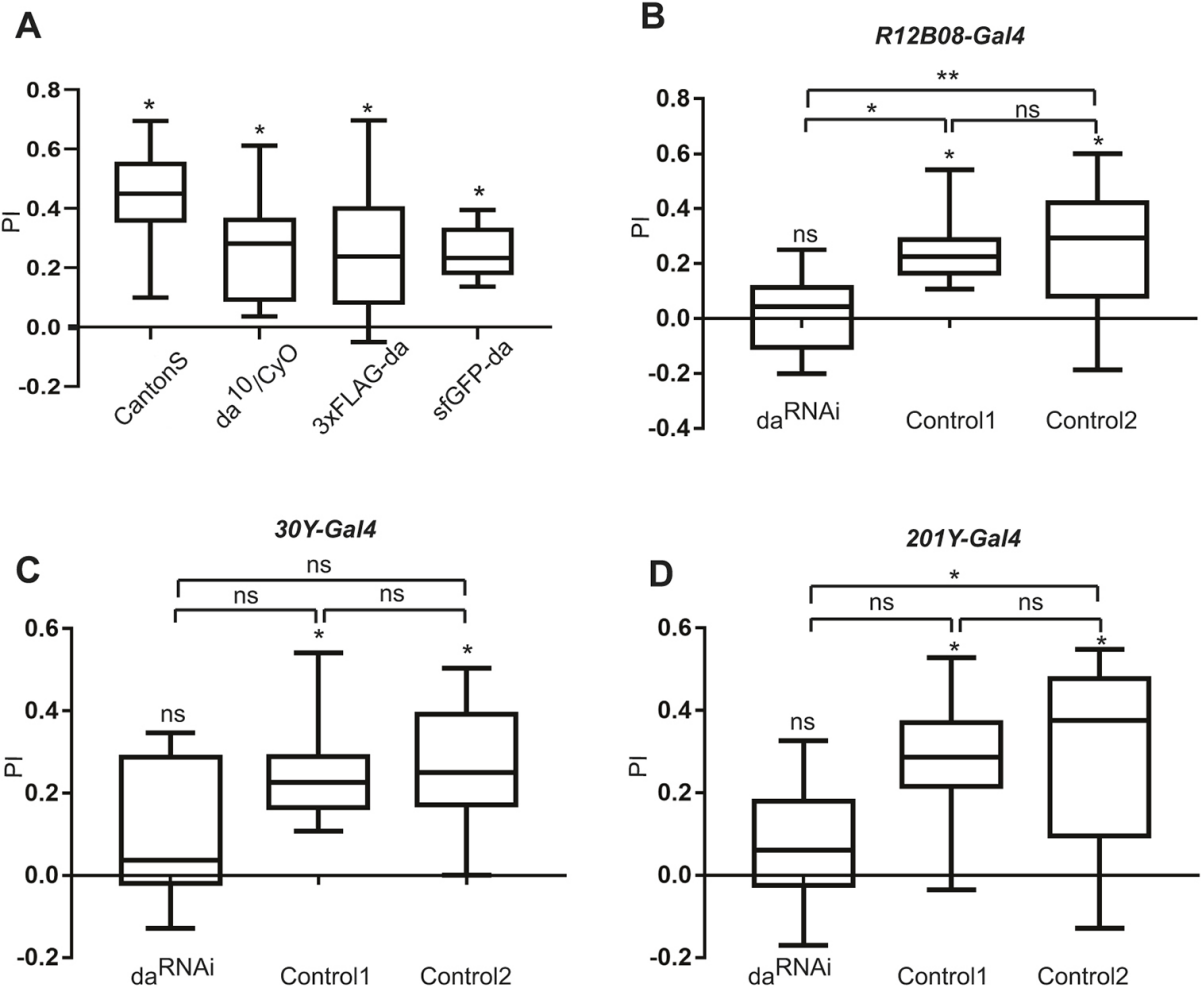
**Knockdown of *da* in the mushroom body leads to impaired olfactory learning of larvae.** (A) CantonS, *da^10^/CyO*, *3xFLAG-da* and *sfGFP-da* larvae have non-zero PI showing appetitive associative memory. (B-D) Larvae have zero PI meaning appetitive associative learning was impaired when *da* was silenced using *R12B08-Gal4* (*UAS-Dcr2;UAS-da^RNAi^ KK105258;R12B08-Gal4* homozygotes) (B), *30Y-Gal4* (*UAS-Dcr2/+;UAS-da^RNAi^ KK105258/+;30Y-Gal4/+* heterozygotes) (C) and 201Y-Gal4 (*UAS-Dcr2;201Y-Gal4;UAS-da^RNAi^ GD51297* homozygotes) (D) indicated by ns over the boxes of da^RNAi^. For Control1 in B, C and D, *UAS-da^RNAi^* larvae were used without drivers. For Control2, *UAS-Dcr2;+;R12B08* homozygotes were used in B, *UAS-Dcr2/+;+;30Y-Gal4/+* heterozygotes in C and *UAS-Dcr2;201Y-Gal4;+* homozygotes in D. PIs are visualized using box-whisker plots that show the median, the 25% and 75% quantiles (boxes), and the minimum to maximum (whiskers). For statistical analysis to determine PI difference compared to zero inside one genotype, a one-sample sign test (asterisks indicated over the boxes) was used. Between the groups, a Kruskal–Wallis ANOVA with Dunn's post-hoc test (indicated on the brackets) was used. *P<0.05, **P<0.01; ns, not significant.

Heterozygous mutations in *TCF4*, the orthologue of *da*, lead to PTHS syndrome, which is characterized by intellectual disability. This fact, and the observation that Da is expressed in a portion of Kenyon cells contributing to the mushroom body, imply that Da might be involved in learning and memory in flies. To test this, we decided to take advantage of the ease of assaying appetitive associative learning and memory in the *Drosophila* larvae (Michels et al., 2017). In this assay, associative memory between odours and taste reward in the larvae was tested. Larvae were trained three times by being presented with one odour with a fructose reward and the other odour with no reward. During the test, larvae were given a choice between the two odours. Then, reciprocal training with a new set of larvae was conducted with subsequent testing. After the second test, the performance index (PI) was calculated. However, this assay showed that learning ability was not impaired in *da* heterozygous mutants *da^10^/CyO* (Fig. 4A), which could be due to *da* upregulation by autoregulation (Smith and Cronmiller, 2001). As sfGFP-Da showed diminished transactivation capability *in vitro* (see above), we also tested homozygous *sfGFP*-*da* larvae and found no impairment of learning (Fig. 4A). Thus, we next investigated whether knockdown of *da* with concurrent enhancement by Dicer-2 (Dcr2) expression (Dietzl et al., 2007) in the *Drosophila* CNS could impact memory and learning ability. To silence *da* in the CNS, we used several CNS-specific *Gal4* lines. We found that silencing of *da* using three drivers, *R12B08-Gal4* (Fig. 4B) and mushroom body-specific lines *30Y-Gal4* (Fig. 4C) and *201Y-Gal4* (Fig. 4D) (genotypes: *UAS-Dcr2;UAS-da^RNAi^;R12B08-Gal4*, *UAS-Dcr2/+;UAS-da^RNAi^/+;30Y-Gal4/+* and *UAS-Dcr2;201Y-Gal4;UAS-da^RNAi^*) caused larvae to have zero PI, meaning their appetitive associative learning was impaired. For controls, we used both the *UAS-da^RNAi^* line and the *UAS*-*Dcr2* driven by the CNS-specific *Gal4* line (genotypes: *UAS-Dcr2;+;R12B08-Gal4*,* UAS-Dcr2/+;+;30Y-Gal4/+ *or* UAS-Dcr2;201Y-Gal4;+*). All of the control larvae had a non-zero PI with regards to memory. In the case of the *UAS*-*Dcr2;3xFLAG-da,UAS*-*da^RNAi^;R12B08-Gal4* line (in which *3xFLAG-da*, *UAS-da^RNAi^* and *R12B08-Gal4* were all in a homozygous state), Da levels in the larval brains were reduced by ∼25% and ∼35% when compared to *UAS-Dcr2;3xFLAG-da;R12B08-Gal4* and *3xFLAG-da* larval brains, respectively (Fig. S2). To validate that the observed learning phenotype was caused by *da* silencing and not by off-target effects, we conducted rescue experiments using simultaneous *R12B08-Gal4*-driven *da* silencing and overexpression. *UAS-Dcr2;UAS-da^RNAi^;R12B08-Gal4,UAS-da/+* larvae had a non-zero PI, whereas *UAS-Dcr2;UAS-da^RNAi^;R12B08-Gal4/+* had a zero PI (Fig. S3), indicating that overexpressing da partially rescued the memory deficit. Larvae were also tested for their ability to taste and smell. Silencing of *da* using *R12B08-Gal4*, *30Y-Gal4* or *201Y-Gal4* did not impair fructose (Fig. S4A), amyl-acetate (AM) (Fig. S4B) or octanol (OCT) preference (Fig. S4C). Interestingly, *UAS-Dcr2;UAS-da^RNAi^;R12B08-Gal4* larvae had a higher preference for odours; however, this preference was cancelled out due to reciprocal training. In addition, in the memory test situation there were two odours present on the Petri dish, but in the smell sensing test there was only one, which could explain why larvae tended to move towards it. All tested larvae moved around the agar plate, which indicated that they had normal locomotion. Our findings suggest that for normal larval appetitive associative memory, appropriate Da levels are needed in the brain structures specified by *R12B08-Gal4*, *30Y-Gal4* and *201Y-Gal4*.

### Reduced levels of Da in the larval CNS lead to decreased expression of synaptic proteins Syn and Dlg1

**Fig. 5.**
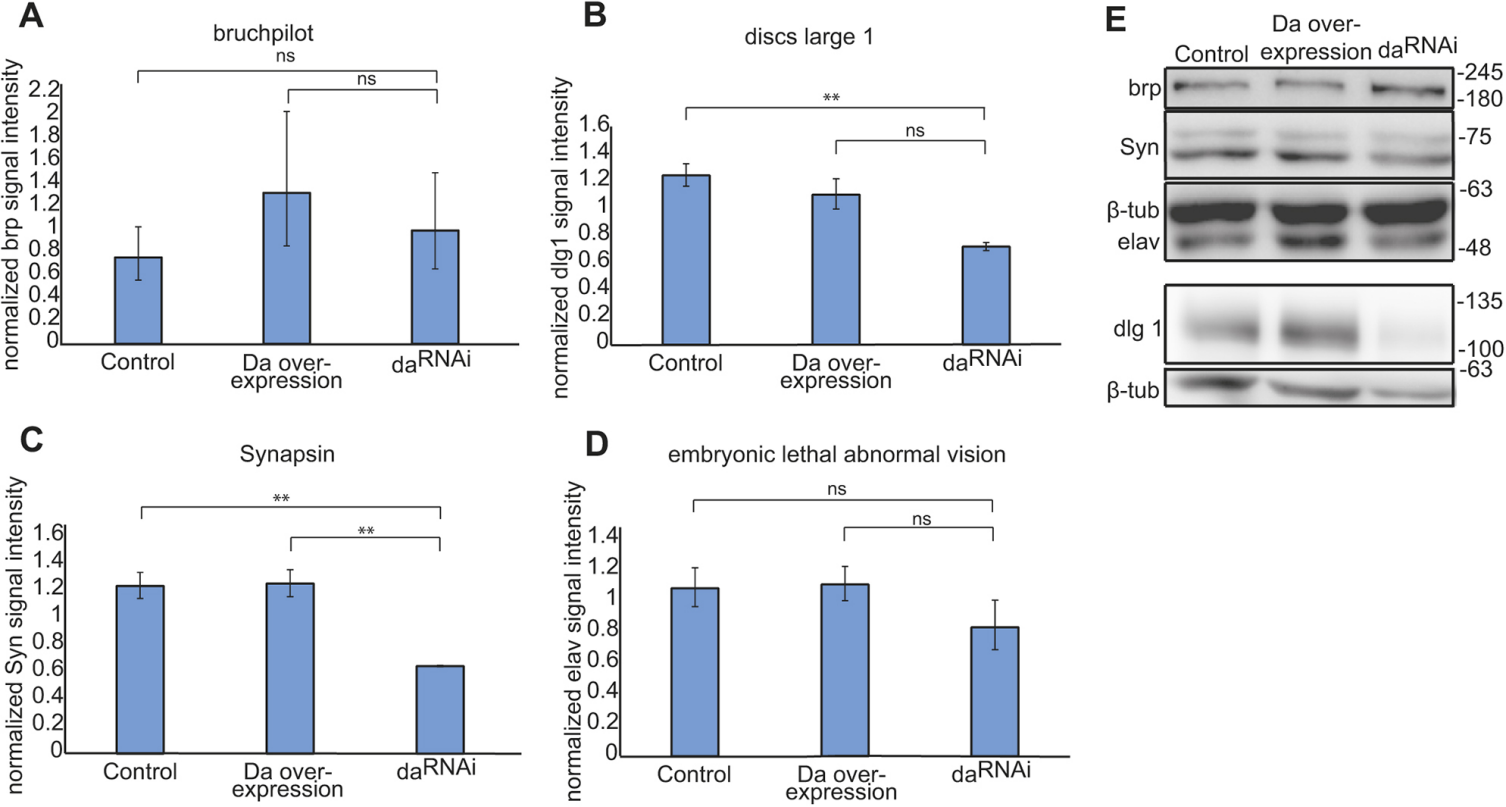
**Silencing of *da* lowers the expression levels of Syn and Dlg1.** Western blot was carried out using larval brains in which *da* was silenced with *R12B08-Gal4*. (A-D) Results of densitometric analysis of the western blot. Protein signals were normalized using β-tubulin signals. The mean results from four independent western blots are shown. Data are mean±s.e.m. Statistical significance is shown with asterisks between the groups connected with brackets. **P<0.01; ns, not significant. Paired, two-tailed Student's *t*-test. Overexpression of Da does not alter bruchpilot, discs large 1, Syn or Elav levels (A-D). Dlg1 and Syn expression levels were lower when Da was silenced (B,C). Silencing of Da did not change bruchpilot and Elav expression levels (A,D). (E) Representative western blot using third instar larval brains. Numbers indicate the molecular weight of proteins in kDa. Control, *R12B08>Dcr2* larval brains; Da overexpression, *R12B08>Da* larval brains; da^RNAi^, *R12B08>Dcr2,da^RNAi^* larval brains.

To investigate the putative mechanisms underlying learning and memory deficits in larvae with lowered levels of Da in the nervous system, we used the driver line *R12B08-Gal4* for silencing *da*, as it had the broadest expression. We compared the expression levels of several known synaptic proteins in the third instar larval brains under both *da* knockdown and overexpression conditions using the *R12B08-Gal4* line (Fig. 5). We quantified the expression levels of the presynaptic protein bruchpilot (Brp) (Fig. 5A), postsynaptic protein Dlg1 (Fig. 5B), presynaptic Syn (Fig. 5C), which is important for learning and memory (Michels et al., 2005), and pan-neuronally expressed neuronal-specific splicing factor embryonic lethal abnormal vision (Elav) (Fig. 5D). We found that the levels of both Dlg1 and Syn were reduced in third instar larval brains with lower levels of Da (Fig. 5B,C,E). On the other hand, Da overexpression did not result in increased levels of these proteins. To further confirm that lowered Da levels decrease Syn and Dlg1 expression we used immunohistochemistry in third instar larval brains. For the silencing of *da*, we used the *201Y-*Gal4 line as it was the strongest and most specific driver in the mushroom body of the lines used. We detected weaker Syn and Dlg1 levels compared to controls (Fig. S5). The levels of Elav and Brp were not significantly changed by knockdown or overexpression of *da* (Fig. 5A,D). The finding that Elav levels were not affected by Da suggests that reducing Da levels does not affect the number of neurons, and the observed learning impairment might instead stem from lowered expression levels of synaptic proteins or, alternatively, from reduced numbers of synapses.

### *Syn* and *dlg1* are Da target genes

**Fig. 6.**
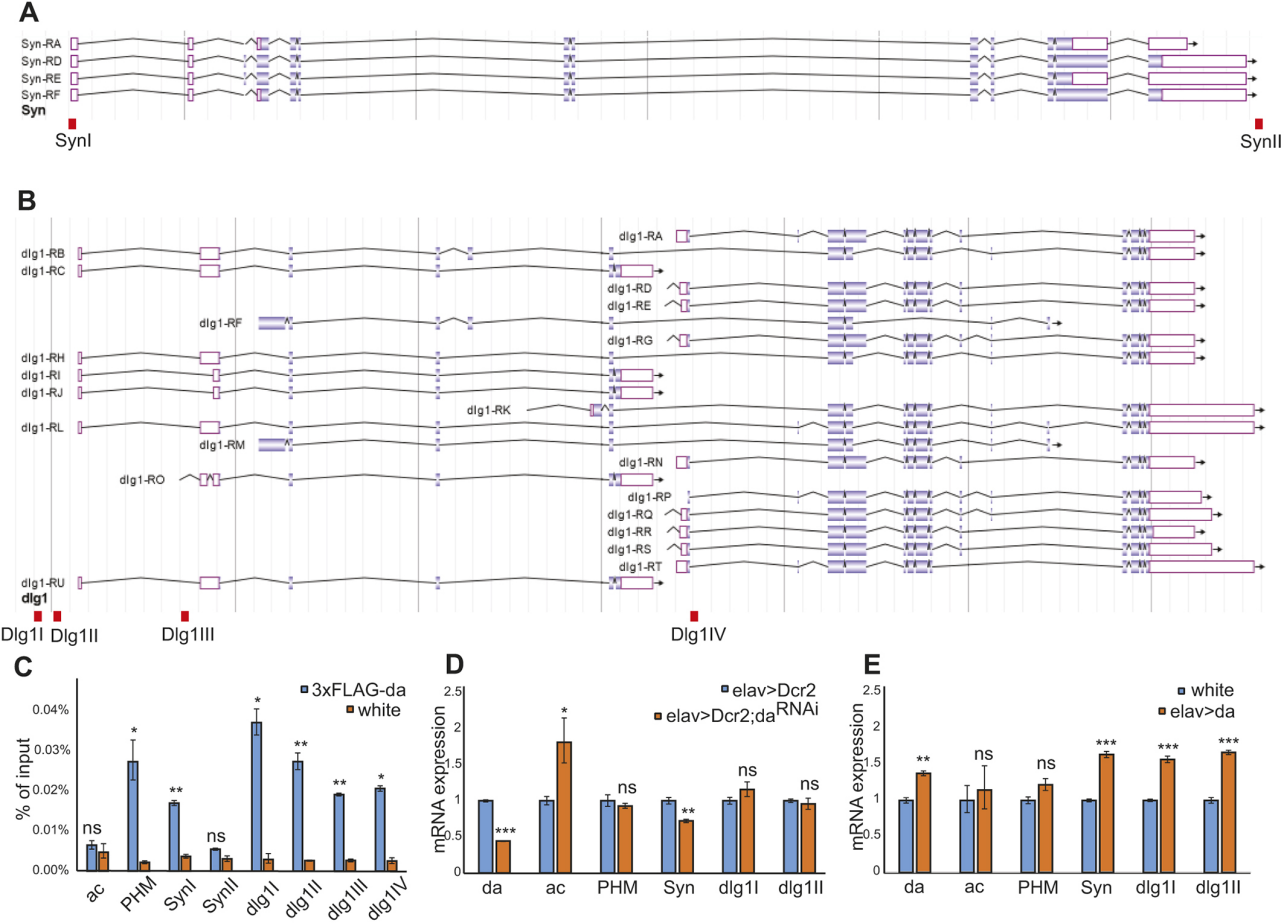
**Da directly regulates *Syn* and *dlg1* in adult fly heads.** (A) JBrowse view of the *Syn* gene. Four annotated transcripts are shown. (B) JBrowse view of the *dlg1* gene. A total of 21 annotated transcripts are shown. Red boxes indicate areas in which primer pairs Syn1, SynII, Dlg1I, Dlg1II, Dlg1III and Dlg1IV amplify DNA. (C) qPCR results from the ChIP experiment with 3xFLAG-*da* and *white^1118^* wild-type adult heads using the anti-FLAG antibody. ac, *achaete* gene locus used as a negative control; PHM, *peptidylglycine alpha-hydroxylating monooxygenase* gene first intron used as a positive control; SynI, promoter region of *Syn*; SynII, 3′ end of *Syn* gene locus; dlg1I, dlg1II, dlg1III and dlg1IV, *discs large 1* gene locus. (D,E) RT-qPCR results showing the effects of *da* silencing (D) or overexpression (E) using *elav-Gal4* on *da*, *ac*, *PHM*, *Syn* and *dlg1* mRNA levels. *da* silencing reduces *da* and *Syn*, and increases *ac* mRNA levels (D). *da* overexpression increases *da*, *Syn* and *dlg1* mRNA levels (E). (C-E) Results from three biological replicates are shown. Data are mean±s.e.m. *P<0.05, **P<0.01, ***P<0.001, Paired, two-tailed Student’s *t*-test.

As Syn and Dlg1 protein levels were reduced in third instar larval brains when *da* was silenced, and it has been shown that Da binds to both *Syn* and *dlg1* gene loci at embryonic stages 4 and 5 (MacArthur et al., 2009), we sought to investigate whether Da binds to these areas in adult heads too. To facilitate this, we conducted a chromatin immunoprecipitation (ChIP) assay in *3xFLAG-da* adult heads using anti-FLAG antibodies. As a control we used the *white^1118^* fly line with no *FLAG* tag. For quantitative PCR (qPCR) from immunoprecipitated chromatin, we designed primers to amplify *Syn* and *dlg1* gene areas containing E-boxes to which Da binds in early embryos (MacArthur et al., 2009). In addition to the previously shown Da-binding site in the *Syn* gene, we also tested Da binding to the *Syn* promoter region (Fig. 6A). For *dlg1*, we designed four primer pairs, as Da has been shown to bind four areas in that gene (Fig. 6B) (MacArthur et al., 2009). As a negative control, we used primers for *achaete* (Andrade-Zapata and Baonza, 2014), as it encodes a proneural protein essential for neuronal development and should not be expressed in adult heads. As a positive control, we used the *peptidylglycine-α-hydroxylating monooxygenase* (*Phm*) gene first intron in which Da binds as a heterodimer with dimmed to activate transcription (Park et al., 2008). qPCR with immunoprecipitated chromatin using *Syn* primers resulted in the enrichment of the *Syn* promoter area (primer pair SynI), whereas the previously reported Da-binding site was not enriched in adult heads (primer pair SynII) (Fig. 6C). All *dlg1* primers resulted in the enrichment of previously reported Da-binding areas (Fig. 6C). This means that Da does not bind to the locus at the 3′ end of the *Syn* gene but binds to the *Syn* promoter and all four *dlg1* gene areas that we selected in the adult heads. To validate *Syn* and *dlg1* as Da target genes, we carried out RT-qPCR analysis in adult *Drosophila* heads under *da* silencing and overexpression conditions. Here, we used pan-neuronal *elav-Gal4* to silence *da* in all neurons. Although upon *da* silencing using *elav-Gal4*, only *Syn* mRNA levels were decreased (Fig. 6D), *da* overexpression using *elav-Gal4* increased mRNA levels of *Syn* and *dlg1* (Fig. 6E). This indicates that both *Syn* and *dlg1* are direct targets of Da in the *Drosophila* nervous system.

### Suppressing Da using *30Y-Gal4* leads to impaired negative geotaxis of adult flies

**Fig. 7.**
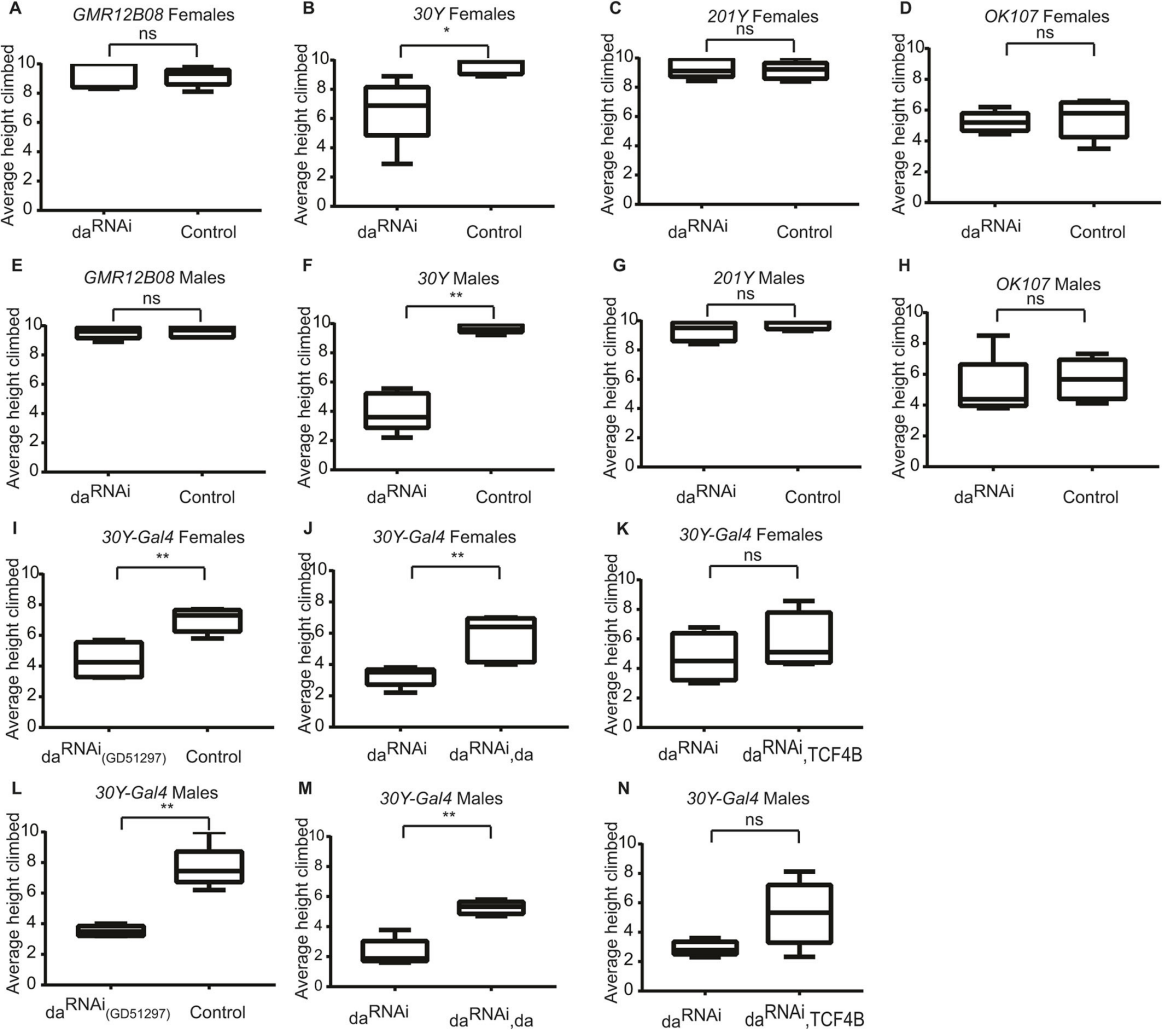
**Silencing of *da* with *30Y-Gal4* impairs negative geotaxis in adult flies.** (A-N) Negative geotaxis was not affected when Da was suppressed using *R12B08-Gal4* (A,E), *201Y-Gal4* (C,G) or *OK107-Gal4* (D,H). The climbing height of the flies was significantly lower when Da was silenced using *30Y-Gal4* (B,F,I,L). (A-N) da^RNAi^, da silencing using *UAS-da^RNAi^ KK105258* (A,B,D-F,H,J,K,M,N) or *UAS-da^RNAi^ GD51297* (C,G,I,L). Control, expressing only Dcr2. (J,M) Overexpression of *da*-rescued geotaxis. (K,N) Expression of human *TCF4B* had a moderate positive effect on geotaxis. Average climbing heights are visualized using box-whisker plots that show the median, the 25% to 75% quantiles (boxes) and the minimum to maximum (whiskers). For statistical significance, Mann–Whitney U tests were used. *P<0.05, **P<0.01; ns, not significant.

Negative geotaxis has been successfully used to evaluate climbing ability indicative of motor dysfunction in the *Drosophila* model for Angelman syndrome, which has similar symptoms to PTHS (Wu et al., 2008). Thus, we next used this assay to evaluate locomotion in adult flies in which *da* knockdown had been achieved by the same drivers as used for the larval learning test. We found that negative geotaxis was unchanged in homozygotes in which *da* knockdown had been achieved by the larval broad neuronal driver *R12B08-Gal4* or mushroom body-specific driver *201Y-Gal4* (Fig. 7A,C,E,G). Interestingly, both female and male heterozygotes in which *da* was silenced by the *30Y-Gal4* driver had severely impaired negative geotaxis (Fig. 7B,F). Rescue experiments were performed to validate that impaired negative geotaxis was caused by *da* silencing and not by off-target effects. The negative geotaxis phenotype was rescued using simultaneous *30Y-Gal4*-driven *da* silencing and overexpression (Fig. 7J,M). Expression of human *TCF4B* under *da-*silencing conditions had a tendency to improve geotaxis (Fig. 7K,N). To further eliminate the possibility of off-target effects, we used the alternative *da^RNAi^* fly line, *UAS-da^RNAi^(GD51297)*, which also caused impairment of negative geotaxis (Fig. 7I,L). An alternative mushroom body driver, *OK107-Gal4*, was used to investigate whether the impairment of geotaxis was caused by lowered Da levels in the mushroom body, but the results revealed no change in negative geotaxis compared to controls (Fig. 7D,H). Next, we visualized Da expression in the adult brains using the *3xFLAG-da* line. Da was expressed widely in the adult *Drosophila* brain including the central brain and thoracic ganglion (Fig. S6), and co-expressed with *30Y-Gal4* in many Kenyon cells in the mushroom body (Fig. S6A-A″). Fewer *OK107-Gal4*^+^ cells were also Da^+^ (Fig. S6B-B″). Cells that cause negative geotaxis impairment when *da* is silenced must be marked by *30Y-Gal4* and not by *OK107-Gal4*, as silencing *da* by *30Y-Gal4* but not by *OK107-Gal4* caused negative geotaxis impairment. *30Y-Gal4* has broader expression outside the mushroom body; for example, in the thoracic ganglion. Silencing *da* using *R12B08-Gal4* or *201Y-Gal4* did not result in impaired negative geotaxis, possibly because co-expression of the drivers and Da is limited in the adult *Drosophila* brain (Fig. S6C-D″).

### Larval appetitive associative learning and negative geotaxis assays can be used for screening drugs for PTHS treatment

**Fig. 8.**
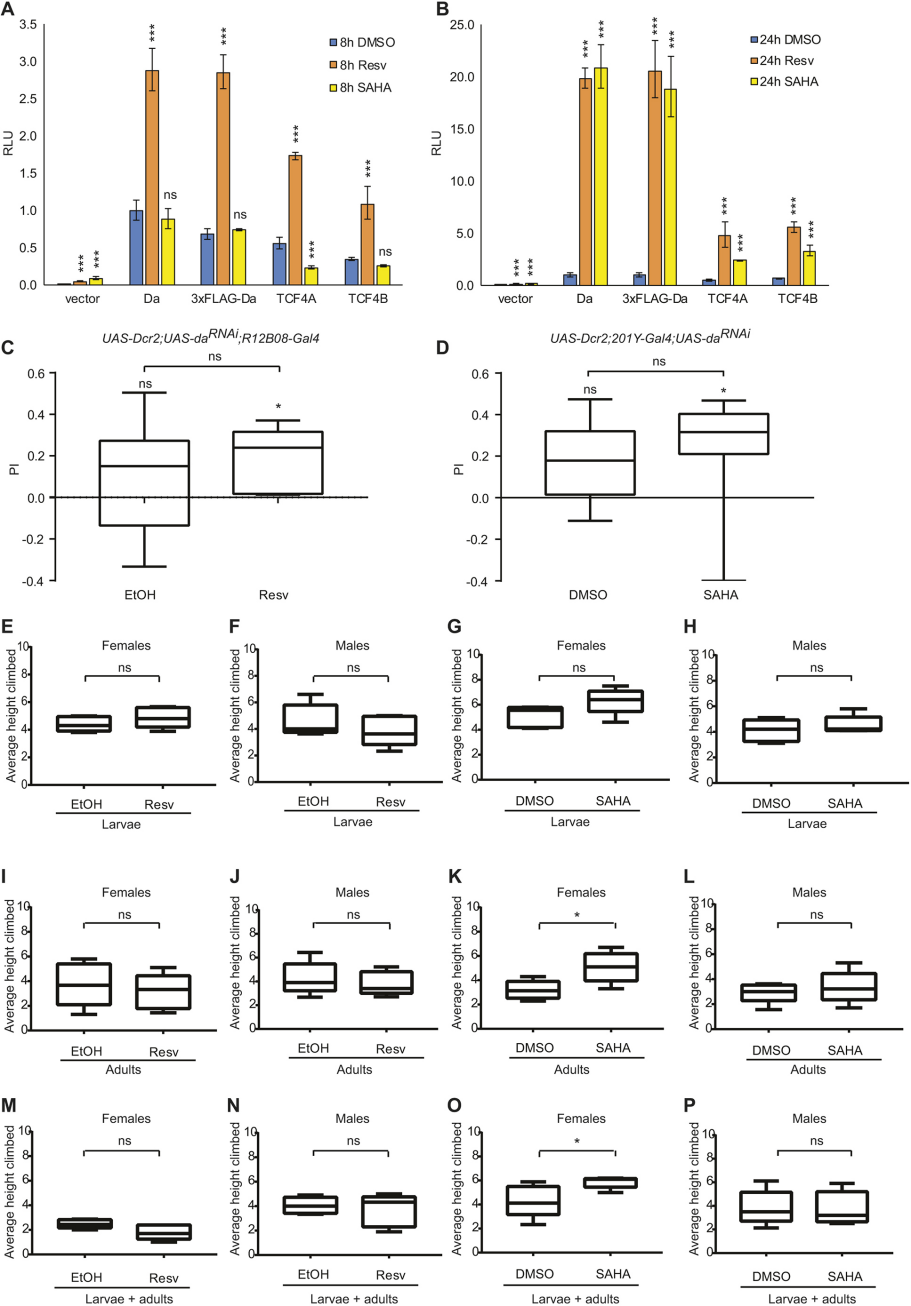
**Resveratrol and SAHA have moderate positive effects on rescuing the impaired learning and negative geotaxis phenotype resulting from decreased levels of Da.** (A,B) Cultured rat primary neurons were co-transfected with constructs encoding wild-type *da*, 3x-*FLAG-tagged da*, empty vector, *TCF4A* or *TCF4B*, a firefly luciferase construct carrying 12 µE5 boxes with a *TK* promoter, and a *Renilla* luciferase construct with mouse *Pgk* promoter for normalization, and treated with 50 μM resveratrol or 5 μM SAHA. Luciferase activities were measured and data are presented relative to the luciferase signals obtained from cells transfected with the wild-type *da-*encoding construct and treated with 0.1% DMSO for control. Mean results from three independent transfection experiments performed in duplicates are shown. Data are mean±s.e.m. Statistical significance is shown compared to 0.1% DMSO-treated cells expressing the respective effector protein. ***P<0.001; ns, not significant; one way ANOVA with post-hoc Bonferroni test; RLU, relative luciferase unit. (C,D) In the larval appetitive associative learning paradigm, the addition of 400 μM resveratrol or 2 μM SAHA to the growth media improved memory. (E-P) In the negative geotaxis assay 400 μM resveratrol or 2 μM SAHA was added to the food substrate of *UAS-Dcr2/+;UAS-da^RNAi^/+;30Y-Gal4/+* only during larval development (E-H), during 5 days after eclosion (I-L) or both during larval development and 5 days after eclosion (M-O). Impaired negative geotaxis was improved when SAHA was fed to females only during adulthood (K) or both during larval development and after eclosion (O). In C-P, results are shown as box-whisker plots that show the median, the 25 to 75% quantiles as boxes and the minimum to maximum as whiskers. For statistical analysis, a one-sample sign test was used, as indicated above the boxes, and a Mann–Whitney U test was used indicated above the brackets. ns above the boxes in C and D indicates zero PI, * indicates non-zero PI, ns above the brackets indicates not significant and *P<0.05 (above the brackets).

Our finding showing that larval appetitive associative learning and adult negative geotaxis become impaired upon *da* silencing, indicates that these fly lines could be used for modelling certain aspects of PTHS in *Drosophila* and testing potential drug candidates. For example, various drugs or drug candidates could be tested for their capacity to rescue these behavioural impairments. As TCF4-dependent transcription is activated by cAMP-PKA pathway-mediated phosphorylation of TCF4 in mammals (Sepp et al., 2017), and resveratrol inhibits cAMP-degrading phosphodiesterases, which leads to elevated cAMP levels (Park et al., 2012), we sought to investigate whether resveratrol increases the transactivational capability of Da and TCF4. We also tested the histone deacetylase inhibitor suberoylanilide hydroxamic acid (SAHA), as it has been shown to rescue memory impairment in the mouse model of PTHS (Kennedy et al., 2016). Therefore, we used the luciferase reporter system in cultured rat primary cortical neurons. Treating neurons with resveratrol for 8 h and resveratrol or SAHA for 24 h significantly increased the E-box*-*dependent transactivational capability of Da, 3xFLAG-da and two human TCF4 isoforms, a shorter isoform TCF4A and a longer isoform TCF4B (Sepp et al., 2011) (Fig. 8A,B). To validate that the increase in luciferase signals seen after treatments with resveratrol and SAHA was caused by an increase in the transcriptional activity of Da, TCF4A and TCF4B, and not by other effects, we performed luciferase reporter assays with Da, TCF4A and TCF4B mutants bearing mutations in the bHLH domain (Fig. S7). Previously, it has been shown that mutations in the bHLH domain abolish transcriptional activity of Da, TCF4A and TCF4B (Forrest et al., 2012; Sepp et al., 2012; Tamberg et al., 2015). Our results confirmed that mutations in the bHLH domain cause loss of transcriptional activity of Da, TCF4A and TCF4B. Next, we decided to test these two substances in the appetitive associative learning and negative geotaxis experiments. We observed that although the *da* knockdown larvae fed with 400 μM resveratrol or 2 μM SAHA showed increased associative memory as their median PI was significantly different from zero, i.e. non-zero PI, the rescue of the learning deficit was not significantly different compared to the controls (Fig. 8C,D). We also tested negative geotaxis of *da* knockdown by *30Y-Gal4* flies fed with 400 μM resveratrol or 2 μM SAHA during larval development (Fig. 8E-H) and 5 days after eclosion (Fig. 8I-L), and both treatments during larval development and 5 days from the beginning of adulthood (Fig. 8M-P). SAHA improved the impairment of negative geotaxis of female flies when they were fed after eclosion (Fig. 8K) or during larval development and after eclosion (Fig. 8O). In addition, our results showed that resveratrol and SAHA did not increase TCF4 and Da protein levels (Fig. S8A-D), confirming that the activation of E-box-controlled reporter genes was caused by an increase in transcriptional activity of TCF4 and Da, and not by an increase of their protein levels. Next, we investigated whether feeding resveratrol or SAHA to larvae causes an increase in Da targets Syn and Dlg1, but we were unable to detect any increase in the expression of these proteins (Fig. S8E-G). These results indicate that the improvement of the learning and geotaxis phenotypes by resveratrol and SAHA are possibly caused by other mechanisms rather than rescuing the levels of synaptic proteins Syn and Dlg1. Nevertheless, rescuing negative geotaxis impairment caused by lowered levels of Da provides a powerful tool for finding drugs that can potentially improve PTHS symptoms.

## DISCUSSION

Here, we characterized the expression of Da protein in the *Drosophila* larval and adult brain. Da was expressed in many areas of the brain, including populations of Kenyon cells in the mushroom body, which is the centre for learning and memory in the fruit fly and carries out a role that is comparable to the mammalian hippocampus. Single-cell RNA sequencing has shown that *da* is expressed widely in the adult brain and that *da* co-expresses with *eyeless* and *portabella*, which are markers for mushroom body Kenyon cells (Davie et al., 2018). The orthologue of *da*, *TCF4*, is expressed not only in the adult mammalian hippocampus but also in cortical and subcortical structures (Jung et al., 2018).

We created N-terminally tagged *3xFLAG-da* and *sfGFP-da* fly strains. Both strains are homozygous viable and fertile, indicating that the overall functionality of Da *in vivo* is not altered by the molecular tag. However, in a luciferase reporter assay in mammalian HEK293 cells, the sfGFP tag reduced transcription activation capability of Da. E-proteins activate transcription preferably as heterodimers with class II bHLH proteins, but can also act as homodimers (Cabrera and Alonso, 1991). In mammalian HEK293 cells, Da probably activates transcription as a homodimer, as Da levels are high due to overexpression, so homodimer formation is preferred (Sepp et al., 2012). This suggests that the sfGFP tag could interfere with Da function as a homodimer in the luciferase assay but not as a heterodimer *in vivo*. We also compared the appetitive associative learning ability of *3xFLAG-da* and *sfGFP-da* larvae, and both of the lines had no learning impairment in this assay. This provides additional evidence that the 3xFLAG tag does not affect Da function and the sfGFP tag reduces its transactivational capability, probably by interfering with Da homodimer function*.*

As Da is widely expressed in the third instar larval CNS with some expression in structures associated with learning and memory, and PTHS is caused by heterozygous mutations in *TCF4*, we tested the learning ability of *da* heterozygous mutant larvae. These larvae had no memory impairment, which could be because of *da* upregulation by autoregulation (Smith and Cronmiller, 2001). Learning and memory of the larvae were impaired when *da* was knocked down using a broad neuronal driver and two mushroom body-specific drivers, although in third instar larval brains Da co-expression with the mushroom body drivers was limited. The impaired learning phenotype could be explained by neurodevelopmental issues resulting from lowered levels of Da protein during development or, alternatively, by the contribution of cells outside the mushroom body. Da mammalian orthologue TCF4 is also associated with learning and memory, as when *TCF4* is downregulated in the mouse hippocampus, pathways associated with neuronal plasticity are dysregulated (Kennedy et al., 2016), and silencing of *TCF4* in human pluripotent stem cell-derived neurons results in downregulated signalling pathways that are important for learning and memory (Hennig et al., 2017). In *TCF4* conditional knockout mice, the neurons in the cortex and hippocampus have reduced numbers of dendritic spines, which also suggests that synaptic plasticity is altered (Crux et al., 2018). In multiple PTHS mouse models, spatial learning is defective probably as a result of hippocampal N-methyl-D-aspartate receptor (NMDA) hyperfunction (Thaxton et al., 2018). Furthermore, many genes that code for synaptic proteins and have been linked to autism, intellectual disability, or psychiatric diseases, are direct targets of TCF4 (Forrest et al., 2018; Hennig et al., 2017).

Here, we showed that when *da* was silenced using a driver with broad expression in the *Drosophila* larval brain, expression levels of synaptic proteins Dlg1 and Syn was downregulated. Dlg1 is a member of the membrane-associated guanylate kinase (MAGUK) protein family. Several vertebrate homologues of Dlg1 have been shown to be important for learning and memory. Discs large MAGUK scaffold protein 3 [DLG3; also called synapse-associated protein 102 (SAP102)] knockout mice have spatial learning deficit (Cuthbert et al., 2007), and in human *DLG3*, mutations that cause dysfunctional NMDA receptor signalling have been associated with X-linked mental retardation (Tarpey et al., 2004; Zanni et al., 2010). We also found that Da is a direct regulator of *dlg1*, as in adult *Drosophila* heads, Da binds to multiple areas in the *dlg1* gene and *dlg1* expression is upregulated when *da* is overexpressed. The gene coding for discs large MAGUK scaffold protein 2 [DLG2; also called postsynaptic density protein 93 (PSD-93)], which is a homologue of *Drosophila dlg1*, is a direct target of TCF4 (Hennig et al., 2017), which indicates that Da and TCF4 share at least some common mechanisms in regulating learning and memory.

Synapsins are presynaptic phosphoproteins that regulate synaptic output (reviewed by Diegelmann et al., 2013). There are three genes that encode vertebrate Synapsins but only one *Syn* gene in *Drosophila* (Klagges et al., 1996)*.* Using knockout experiments in mice, it has been shown that Synapsins are involved in learning and memory (Gitler et al., 2004; Silva et al., 1996), and *SYN1* has been implicated in human neurological diseases, such as learning difficulties and epilepsy (Garcia et al., 2004). Likewise, *Drosophila syn^97^* mutant larvae have impaired appetitive associative learning (Michels et al., 2005). The fact that the memory of *syn^97^* larvae can be rescued by expressing Syn in the mushroom bodies (Michels et al., 2011), is consistent with our findings that lower Da levels affect Syn expression levels and that appropriate Da levels are required for proper memory formation. Syn-dependent memory is likely formed by its phosphorylation by Protein kinase A (PKA) (Michels et al., 2011). When Syn is phosphorylated at its PKA/CamK I/IV (Protein kinase A/Ca^2+^/calmodulin-dependent protein kinase I/IV) sites, its affinity for actin is reduced and synaptic vesicles from the reserve pool can be exocytosed (reviewed by Benfenati, 2011). We found that Syn is likely a direct target of Da as Da binds to the *Syn* promoter, and both silencing and overexpression of *da* changes *Syn* mRNA levels.

We also sought to rescue the learning phenotype caused by *da* silencing. To facilitate this, we fed the larvae with resveratrol or SAHA, as our luciferase reporter experiments in primary neuronal cultures showed that resveratrol and SAHA significantly improve the transactivational capability of both Da and TCF4. Resveratrol inhibits cAMP-degrading phosphodiesterases, which leads to elevated cAMP levels (Park et al., 2012), and TCF4-dependent transcription upon neuronal activity is activated by cAMP-PKA pathway-mediated phosphorylation of TCF4 (Sepp et al., 2017). It is plausible that Da could also be regulated by phosphorylation by PKA; therefore, resveratrol improves Da transactivational capability. Also, resveratrol had a moderate positive effect on learning and memory in the *da* knockdown larvae. Whether this effect is linked to the cAMP-PKA pathway has yet to be verified. SAHA is a histone deacetylase inhibitor that improves learning and memory in *TCF4*(+/−) mice through the normalization of synaptic plasticity (Kennedy et al., 2016). Here, we showed that feeding SAHA to *Drosophila* larvae also had a moderate effect on learning.

Silencing of *da* by *30Y-Gal4* impaired negative geotaxis. We also sought to rescue the impaired geotaxis of *30Y>Dcr2;da^RNAi^* flies using resveratrol or SAHA. We administered the drugs in the food substrate either during development or to adult flies or at both developmental stages. Negative geotaxis of female flies was significantly improved when SAHA was administered only after eclosion, or during both larval development and after eclosion. Supplementing the food of larvae only had no effect on negative geotaxis of the adults. The finding that SAHA only improved the phenotype of females could be due to the amount ingested by the males not being enough to rescue the geotaxis phenotype caused by lowered levels of Da. Recently, it has been shown that female flies do indeed consume more food than male flies (Wu et al., 2020), which could be the reason for SAHA improving geotaxis impairment of only female flies. In a recent study in *Drosophila*, in which genes associated with autism spectrum disorders and intellectual disability were suppressed, the knockdown of Da resulted in impaired habituation (Fenckova et al., 2019). The rescue of this habituation phenotype could also be tested to examine whether it can be improved with drugs.

Our study demonstrates that the levels of the TCF4 homologue Da are important for memory and negative geotaxis, possibly via regulation of the synaptic proteome. These novel learning and geotaxis deficiency models can be further used for screening therapeutics for TCF4-related diseases. Recently, using deconvolution analysis, TCF4 was identified as a master regulator in SCZ (Doostparast Torshizi et al., 2019). This opens up new avenues for using *Drosophila* to model TCF4-related diseases.

## MATERIALS AND METHODS

### *Drosophila* stocks

All *Drosophila* stocks and crosses were fed with malt and semolina-based food with 12 h light and dark daily rhythms at 25°C with 60% humidity, unless mentioned otherwise. *Drosophila* strains used in this study were *UAS-da^RNAi^* GD51297 and *UAS-da^RNAi^* KK105258 from the Vienna *Drosophila* Resource Center, CantonS (a gift from Dr Bertram Gerber, Leibniz Institute for Neurobiology, Magdeburg, Germany), *da^G32^-Gal4* (a gift from Riitta Lindström, University of Helsinki, Helsinki, Finland), *UAS-TCF4B* (Tamberg et al., 2015), *201Y-Gal4* [Bloomington *Drosophila* Stock Center (BDSC, 4440)], *30Y-Gal4* (BDSC, 30818) (Yao Yang et al., 1995) and *OK107-Gal4* (BDSC, 854) were gifts from Mark Fortini, Thomas Jefferson University, Philadelphia, PA, USA. *R12B08-Gal4* (BDSC, 48489) (Pfeiffer et al., 2008, flweb.janelia.org/cgi-bin/view_flew_imagery.cgi?line=R12B08), *elav-Gal4* (BDSC, 8760) (Luo et al., 1994), *UAS-Dcr2;Pin^1^/CyO* (BDSC, 24644) (Dietzl et al., 2007), *UAS-nls-GFP* (BDSC, 4776), *UAS*-*da^G^* (BDSC, 37291), *UAS-mCD8-GFP; Pin^1^/CyO* (BDSC, 5136) and *da* mutant line *da^10^* (BDSC, 5531) (Caudy et al., 1988) were obtained from the BDSC. The following transgenic lines were generated in this study: *3xFLAG*-*da^2M4^* and *sfGFP*-*da^4M1^*.

### Endogeneous tagging of Da by CRISPR/Cas9

The coding sequence for *3xFLAG-* or *sfGFP-*tag was inserted into the 5′ coding region of the *da* gene using CRISPR/Cas9 technology. The genomic sequence around the tag was as follows: 5′-ATGGCGA**CCA**GTG|ACGATGAGCC-3′ (PAM sequence shown as bold and the cut site marked with |). For the higher mutagenesis rate, a specific fruit fly line for guide RNA production was created. Partially overlapping oligonucleotides, 5′-CTTCGTGCATCGGCTCATCGTCAC-3′ and 5′-AAACTGGACGATGAGCCGATGCAC-3′, designed to target the N-terminus of the Da protein, were cloned downstream of the polymerase III U6:2 promoter in the pCFD2-dU6:2gRNA plasmid (Addgene #49409). Transgenic flies expressing gRNAs were created by injecting the generated plasmid into *PBac{yellow^+^-attP-9A}VK00027* (BDSC, 9744) fly strain embryos. For donor plasmid generation, *pHD-3xFLAG-ScarlessDsRed* or *pHD-sfGFP-ScarlessDsRed* [both were gifts from Kate O'Connor-Giles, *Drosophila* Genomics Resource Center (DGRC), Indiana University, IN, USA] were used with Gibson cloning. The following primer pairs were used for the amplification of upstream and downstream homology arms:

upst F5, 5′-CGGCCGCGAATTCGCCCTTGGTTGTGAATCAGGTGTAGAAACA-3′ and

upst_R, 5′-GCCGGAACCTCCAGATCCACCACTGGTCGCCATTTCAGCA-3′; and

dwns_F, 5′-TTCTGGTGGTTCAGGAGGTTACGATGAGCCGATGCACTTG-3′ and

dwns_R, 5′-GTTTAAACGAATTCGCCCTTAACGCCCTGGAACACCGAGG-3′.

After verification, the obtained donor plasmids *pHD-da-3xFLAG-ScarlessDsRed* and *pHD-da-sfGFP-ScarlessDsRed* were injected into F_1_ embryos from a cross between *da-gRNA* (our gRNA-expressing transgenic strain) and y^1^M{w^+mC^=nos-Cas9.P}ZH-2A w* (BDSC, 54591) fly strains. All embryo injections were ordered from BestGene.

The *dsRed* cassette was removed from selected progeny by crossing to the PiggyBac transposase line *Herm{3xP3-ECFP,αtub-piggyBacK10}M10* (BDSC, 32073) (Horn et al., 2003). The obtained *3xFLAG-da* and *sfGFP-da* lines were verified by sequencing.

### RNA isolation and cloning

RNA from *3xFLAG-da* or *sfGFP-da Drosophila* embryos was isolated using an RNeasy Mini Kit (Qiagen) according to the manufacturer's protocol. cDNA was synthesized using 2 μg of RNA. Primer sequences for cloning were 5′-ACTAGTTGAAGTCGACTGGAC-3′ and 5′-CCAGGTCCTCCAATTCCACC-3′. PCR products containing either *3xFLAG-da* or *sfGFP-da* cDNA sequences were sequenced and cloned into the *pCDNA3.1* expression vector (Tamberg et al., 2015) using BcuI (SpeI, 10 U; Thermo Scientific) and BstII (Eco 91I, 10 U; Thermo Scientific) restriction enzymes. The pcDNA3.1 constructs encoding Da, and reporter vectors pGL4.29[luc2P/12μE5/Hygro], pGL4[hRlucP/min/Hygro], pGL4[hRlucP/PGK/Hygro] and pGL4.29[luc2P/12μE5-TK/Hygro] have been described previously (Sepp et al., 2011, 2012, 2017; Tamberg et al., 2015).

### Cell culture, transfections and luciferase reporter assay

Human embryonic kidney cells HEK-293 were obtained from ATCC (LGC Standards GmbH, Wesel, Germany), routinely tested for contamination and were grown in minimal essential media (Capricorn Scientific) supplemented with 10% fetal bovine serum (PAA Laboratories), 100 U/ml penicillin and 0.1 mg/ml streptomycin (Gibco). For transfection, 0.375 μg of DNA and 0.75 μg of polyethylenimine (Sigma-Aldrich) were used for each well of a 48-well plate, or scaled up accordingly. For co-transfections, equal amounts of pGL4.29[luc2P/12μE5/Hygro], pGL4[hRlucP/min/Hygro] and effector constructs were used. Cells were lysed 24 h after transfection.

Rat cortical neuronal cultures from Sprague Dawley embryonic day (E)22.5 rat embryos were obtained and maintained as described previously (Sepp et al., 2017). All animal procedures were approved by the local ethics committee. Neuronal cultures were transfected at 6 days *in vitro* (DIV) in conditioned medium. For transfection, 120 ng of expression plasmid, 60 ng of pGL4.29[luc2P/12μE5-TK/Hygro], 20 ng of pGL4[hRlucP/PGK/Hygro] and 0.6 μl of Lipofectamine 2000 (Invitrogen) were used. Neurons were treated with resveratrol, SAHA or DMSO as a vehicle and lysed at 8 DIV.

Luciferase assays were performed as described previously (Sepp et al., 2011) using passive lysis buffer (Promega) and the Dual-Glo luciferase assay system (Promega). For data analysis, background signals from untransfected cells were subtracted and firefly luciferase signals were normalised to *Renilla* luciferase signals. The data were then log transformed and auto scaled, means and standard deviations were calculated and paired, two-tailed Student’s *t*-tests were performed. The data were back-transformed for graphical representation.

### Protein electrophoresis and western blotting

For SDS-PAGE, embryos, larvae, pupae, adult heads or larval brains were lysed in 2× SDS sample buffer. Equal amounts of protein were loaded to the gel. The following mouse monoclonal antibodies were obtained from the Developmental Studies Hybridoma Bank (DSHB; University of Iowa, Iowa City, IA, USA): β-tubulin E7 (dilution 1:3000; DSHB AB_2315513, developed by M. Klymkowsky); Synapsin SYNORF1 3C11 (dilution 1:1000; DSHB AB_528479, developed by E. Buchner); Discs large 1 4F3 (dilution 1:2000; DSHB AB_528203, developed by C. Goodman); Elav 9F8A9 (dilution 1:1000; DSHB AB_528217, developed by G. M. Rubin); and Bruchpilot nc82 (dilution 1:100; DSHB AB_2314866, developed by E. Buchner). Other antibodies used were: mouse anti-Da dam109-10 (dilution 1:10; a gift from C. Cronmiller, University of Virginia, Charlottesville, VA, USA); mouse anti-FLAG M2 horseradish peroxidase (HRP)-conjugated (dilution 1:6000; Sigma-Aldrich A8592); and goat anti-mouse IgM HRP-conjugated secondary antibody (dilution 1:5000; Invitrogen 32430).

### Immunohistochemical staining

The anterior parts of third instar larvae were dissected in PBS and fixed using 4% paraformaldehyde in PBS. Adult flies were first fixed in 4% paraformaldehyde in PBS and then dissected. Primary antibody labelling was performed overnight, or for 72 h with the anti-FLAG antibody, on an overhead rotator at 4°C in PBS with 0.1% Triton X-100, or 0.5% Triton X-100 for the anti-FLAG antibody. The antibodies used were as follows: Synapsin SYNORF1 3C11 (dilution 1:10; DSHB AB_528479); Discs large 1 4F3 (dilution 1:400; DSHB AB_528203; mouse anti-FLAG M2 (dilution 1:1000; Sigma-Aldrich F1804); and goat anti-mouse Alexa594 (dilution 1:1000; ImmunoResearch Laboratories 115-585-003). Secondary antibodies were pre-adsorbed to wild-type tissues before use. Incubation with secondary antibodies was performed for 3 h on an overhead rotator at room temperature in PBS with 0.1% Triton X-100. The labelled larval brains were dissected and mounted in Vectashield mounting medium (Vector Laboratories). For image collection, a Zeiss LSM 510 Meta confocal microscope with a Pln Apo 20×/0.8 DICII objective or a Pln Apo 63×/1.4 Oil DICII objective was used. Suitable layers were selected using Imaris software (Bitplane).

### Appetitive associative learning assay

The appetitive associative memory assay in the *Drosophila* larvae was performed as described previously (Michels et al., 2017). Briefly, the larvae were trained three times for 5 min on Petri dishes; the odour-amyl acetate (AM) was presented on plain agar and odour–OCT on agar containing fructose as a reward. Then, the larvae were placed in the midline of a plain agar plate and given a choice between the two odours placed on separate halves of the Petri dish. After 3 min, larvae were counted on each half of the Petri dish. Then reciprocal training was performed with AM and fructose and OCT with plain agar. Using data from two reciprocally trained tests, the PI was calculated PI=(PREF AM_AM+/OCT_−PREF AM_AM/OCT+_)/2. The odours and the reward were presented in four different orders to eliminate any non-specific preferences. Altogether, 12 training and test cycles were conducted per genotype, each time with new larvae, and PIs were calculated and used for statistical analysis. The PIs were visualized as box-whisker plots that showed the median, the 25% and 75% quantiles and the minimum and maximum. For statistical analysis inside one genotype, a one-sample sign test was applied with an error threshold smaller than 5% and between the groups Kruskal–Wallis ANOVA with Dunn's post-hoc test was used. SAHA was dissolved in dimethyl sulfoxide (DMSO) and the same concentration of DMSO (0.1%) was used in the food substrate for a control. Resveratrol was dissolved in 96% ethanol and 1% ethanol in the food that was used for the control.

### ChIP

Chromatin preparations were carried out as described previously (Chanas et al., 2004). Adult heads (∼150 mg) were collected on dry ice and homogenized in buffer A1 [60 mM KCl, 15 mM NaCl, 4 mM MgCl_2_, 15 mM HEPES (pH 7.6), 0.5% Triton X-100, 0.5 mM DTT, 10 mM sodium butyrate and 1× EDTA-free protease inhibitor cocktail (Roche)] with 1.8% formaldehyde at room temperature using a Kontes pellet pestle followed by three strokes using a Dounce homogenizer with a loose pestle. Homogenate was incubated for 15 min and glycin was added to 225 mM of the homogenate followed by 5 min incubation. The homogenate was then centrifuged for 5 min at 4000 ***g*** at 4°C and the supernatant was discarded. The pellet was washed three times with 3 ml of buffer A1 followed by a wash with 3 ml of lysis buffer [14 mM NaCl, 15 mM HEPES (pH 7.6), 1 mM EDTA, 0.5 mM EGTA, 1% Triton X-100, 0.5 mM DTT, 0.1% sodium deoxycholate, 0.05% SDS, 10 mM sodium butyrate and 1× EDTA-free protease inhibitor cocktail (Roche)]. Crosslinked material was resuspended in 0.5 ml of lysis buffer with 0.1% SDS and 0.5% N-lauroylsarcosine, and incubated for 10 min at 4°C on a rotator followed by sonication using a Sonics Vibra-Cell processor at 70% amplitude for 30 times at 15 s intervals. Crosslinked material was then rotated for 10 min at 4°C and centrifuged for 5 min at room temperature at maximum speed. The supernatant was then transferred to a new tube and 0.5 ml of lysis buffer was added to the pellet followed by rotation and centrifugation. Supernatants were combined and centrifuged two times for 10 min each time at maximum speed. Chromatin extract was transferred to Microcon DNA Fast Flow Centrifugal Filter Units (Merck Millipore), blocked with 1 mg/ml bovine serum albumin in PBS, and purified using lysis buffer. The volume of chromatin extract was brought to 1 ml using lysis buffer. Protein concentrations were determined using a bicinchoninic acid assay (Pierce).

After removing equal amounts of inputs, chromatin extracts were diluted 10× using dilution buffer [1% Triton X-100, 150 mM NaCl, 2 mM EDTA (pH 8.0), 20 mM Tris-HCl (pH 8.0) and 1× EDTA-free protease inhibitor cocktail (Roche)] and added to 50 µl of Dynabeads Protein G (Invitrogen) beads that were previously incubated with 5 µg of monoclonal anti-FLAG M2 antibody (Sigma-Aldrich F1804) in 400 μl of 0.05% PBS Tween 20 overnight (antibody dilution 1:80). ChIP was carried out overnight at 4°C. Beads with chromatin were then washed in wash buffer [1% Triton X-100, 0.1% SDS, 150 mM NaCl, 2 mM EDTA (pH 8.0), 20 mM Tris-HCl (pH 8.0) and 1× EDTA-free protease inhibitor cocktail (Roche)] using a magnetic rack for 10 min for three times at 4°C on a rotator, followed by final wash with wash buffer [1% Triton X-100, 0.1% SDS, 500 mM NaCl, 2 mM EDTA (pH 8.0), 20 mM Tris-HCl (pH 8.0) and 1× EDTA-free protease inhibitor cocktail (Roche)]. Chromatin was eluted using three aliquots of 50 µl elution buffer (1% SDS, 100 mM NaHCO_3_ and 1 mM EDTA) for 10 min each time at 37°C. The volume of inputs was brought to 150 µl with elution buffer. For decrosslinking, 8 µl of 5 M NaCl was added and the samples were incubated at 65°C overnight. Then, 2 µl of RNase A (10 mg/ml) was added and the samples were incubated at 37°C for 30 min, followed by incubation with 2 µl of EDTA (0.5 M) and 4 µl Proteinase K (10 mg/ml) at 45°C for 30 min. DNA was extracted using a QIAquick PCR Purification Kit (Qiagen).

### qPCR

For RT-qPCR, 15 heads were collected from 2- to 3-day-old adult flies on dry ice. RNA was extracted using an RNeasy Mini Kit (Qiagen). cDNA was synthesized with Superscript IV Reverse Transcriptase (Invitrogen) and oligo(dT)_20_ primers. qPCR was performed using a LightCycler 480 II (Roche) with Hot FIREPol EvaGreen qPCR Mix Plus (Solis Biodyne). Primer sequences are shown in Table S1.

### Negative geotaxis assay

Ten females and males were separated in fresh vials 48 h before the assay to allow recovery from anaesthesia. Before the test, males and females from control and *da* silencing groups were transferred to empty vials without anaesthesia, which were closed with another upside down vial using sticky tape. The flies were knocked down three times on the table and a photo was taken after 10 s. The height of the vial was divided into ten equal parts and the number of flies in each compartment was counted, and average height was calculated. The experiment was repeated five times, each time with new flies. Average climbing heights were visualized using box-whisker plots that showed the median, the 25% to 75% quantiles and the minimum and maximum. For statistical significance, pairwise Mann–Whitney U tests were used.

## Supplementary Material

Supplementary information
