# Supplementary material for: Daughterless, the Drosophila orthologue of TCF4, is required for associative learning and maintenance of the synaptic proteome
Source: Dis Model Mech. 2020 Jul 30;13(7):dmm042747. doi: 10.1242/dmm.042747 (PMC7406316; doi:10.1242/dmm.042747)
Supplement: Supplementary information [file dmm-13-042747-s1.pdf]

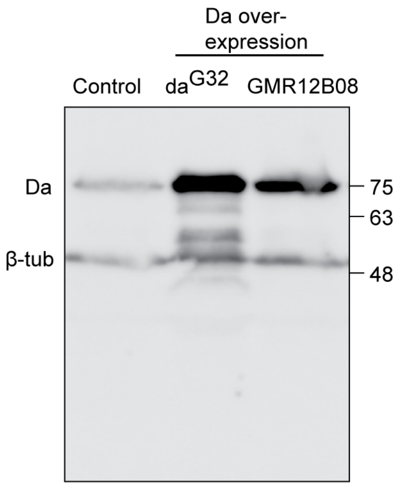

Figure S1. Da overexpression in embryos using ubiquitous driver *da^{G32}-Gal4* reveals additional signals using anti-Da antibody. Western blot analysis of embryos with anti-Da antibody dam109-10, β-tubulin (β-tub) was used as loading control. Control embryos are *GMR12B08>mCD8-GFP*. Additional Da protein forms appear when Da is overexpressed using ubiquitous strong driver *da^{G32}-Gal4* but are not detected using nervous system specific *GMR12B08-Gal4.*

Disease Models & Mechanisms • Supplementary information

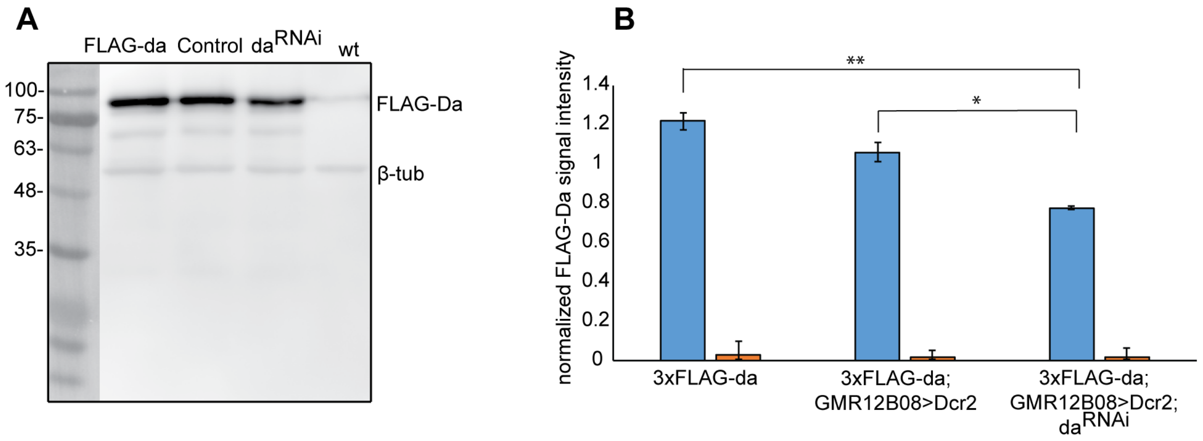

Figure S2. Silencing of *da* in larval brain reduces Da levels. A - Western blot analysis with anti-FLAG antibody of dissected third instar larval brains. Numbers on the left indicate molecular weight in kDa, 3xFLAG-da - larval brains where Da is tagged with 3xFLAG epitope, Control – *3xFLAG-da*;GMR12B08>*Dcr2* larval brains, da^{RNAi} – *3xFLAG-da*;*GMR12B08>Dcr2*,da^{RNAi} larval brains, wt - $white^{1118}$ larval brains for control lacking the 3xFLAG tag. B - upon silencing of *da* the Da expression levels were reduced about 35% compared to *3xFLAG-da* and about 25% compared to *3xFLAG-da;GMR12B08>Dcr2* larval brains. Shown are the results of densitometric analysis of Western blot, 3xFLAG-Da signals were normalized using β-tubulin signals. The mean results from three independent Western blots are shown, blue bars represent mean intensity of 80 kDa protein signal and orange bars represent mean intensity of 65 kDa signal. Error bars show standard error of the mean (s.e.m). Statistical significance is shown with asterisks between the groups connected with lines. *P<0.05, **P<0.01, One Way ANOVA with post-hoc Bonferroni test.

Disease Models & Mechanisms • Supplementary information

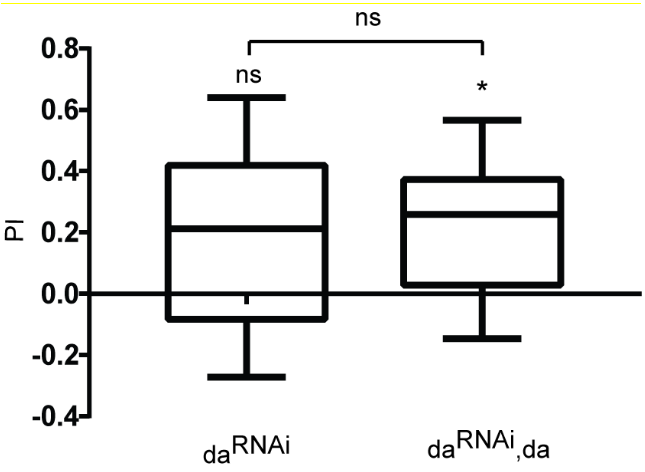

Figure S3. Overexpressing *da* has moderate positive effect on memory deficit caused by silencing of *da*. *UAS-Dcr2;UAS-da^{RNAi};12B08-Gal4/+* - da^{RNAi} – larve zero PI while *UAS-Dcr2;UAS-da^{RNAi};12B08-Gal4,UAS-da/+* - da^{RNAi},da – larve have non-zero PI. PI-s are visualized using box-whisker plots, which show the median, the 25% - 75% quantiles (boxes), and the minimum to maximum (whiskers). To determine PI difference compared to zero inside one genotype one-sample sign test was used (asterisks indicated over the boxes) and between the groups Mann-Whitney U-test was used in statistical analysis.

Disease Models & Mechanisms • Supplementary information

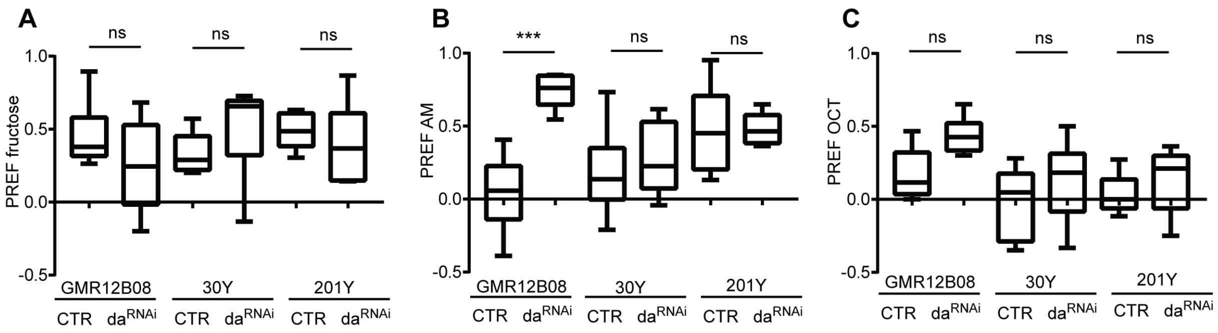

Figure S4. Lowered levels of Da in the nervous system do not impair taste and smell sensing. *da* silencing using *GMR12B08-Gal4*, *30Y-Gal4* or *201Y-Gal4* does not affect fructose tasting (A). *da* silencing using *30Y-Gal4* or *201Y-Gal4* does not affect amyl-acetate (AM) preference, but da silencing using *GMR12B08-Gal4* increases the preference (B). *da* silencing using *GMR12B08-Gal4*, *30Y-Gal4* or *201Y-Gal4* does not affect octanol (OCT) preference (C). For statistical analysis Kruskal Wallis ANOVA with Dunn's post-hoc tests were used. *** p<0.001, ns – not significant.

Disease Models & Mechanisms • Supplementary information

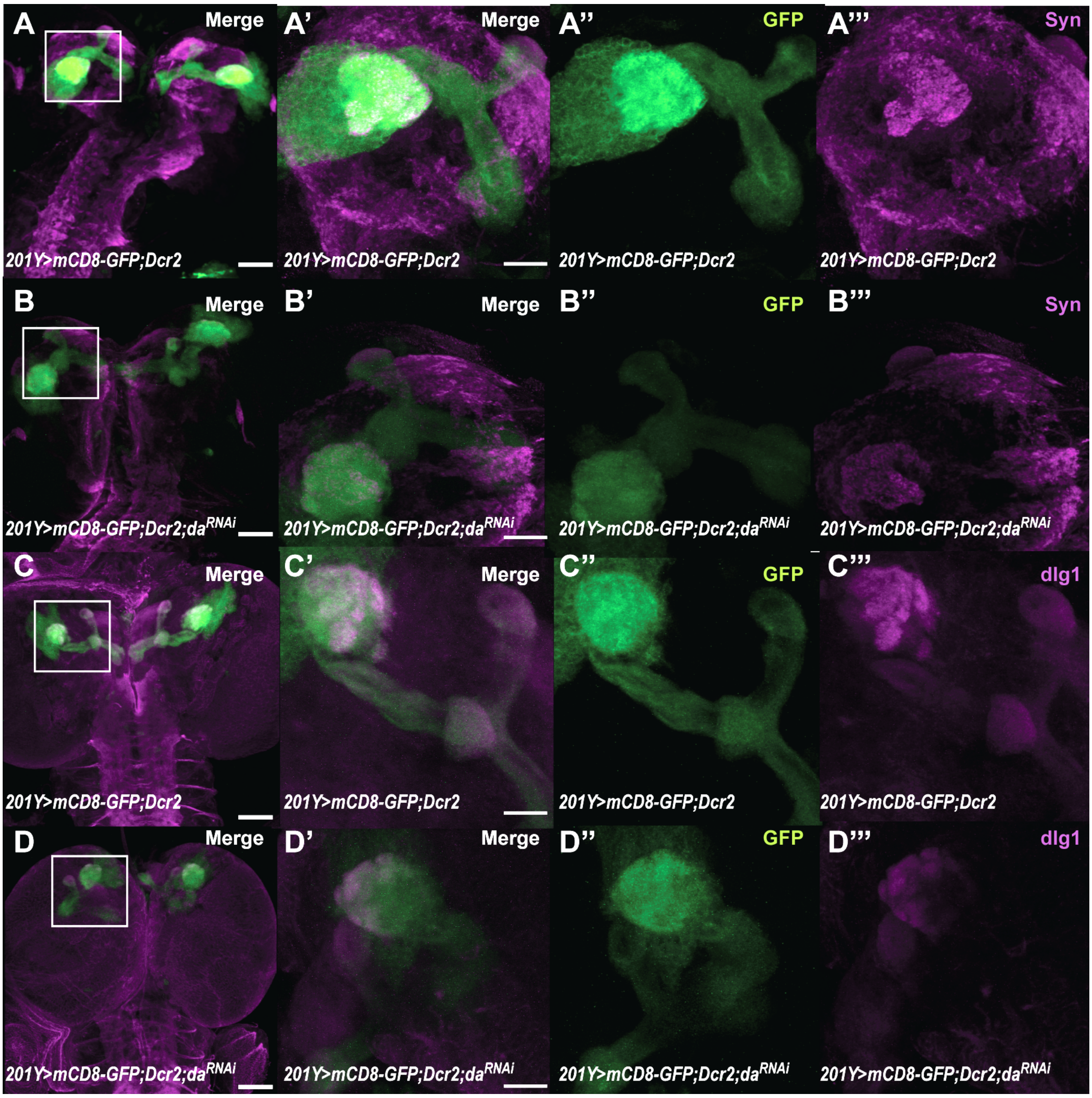

Figure S5. Silencing of *da* by *201Y-Gal4* lowers Syn and dlg1 expression. A-B''' - Immunohistochemical staining of 3rd instar larval brains using anti-Syn antibody reveals that compared to the controls (A-A''') (genotype *UAS-mCD8-GFP/UAS-Dcr2;201Y-Gal4;*+) Syn expression is weaker when *da* is silenced (B-B''') (genotype *UAS-mCD8-GFP/UAS-Dcr2;201Y-Gal4;UAS-da^{RNAi}*). C-D''' - Immunohistochemical staining of 3rd instar larval brains using anti-dlg1 antibody reveals that compared to the controls (C-C''') dlg1 expression is weaker when *da* is silenced (D-D'''). A'', B'', C'', D'' – mCD8-GFP expression shows driver expression pattern. A''' and B'''- expression of Syn, C''' and D''' – expression of dlg1. Scale bars on A, B, C and D represent 50 µm and on A', B', C' and D' 20 µm. The same microscope settings were used for controls and *da*-silenced brains. Larvae were kept at 30˚C for stronger *da* silencing.

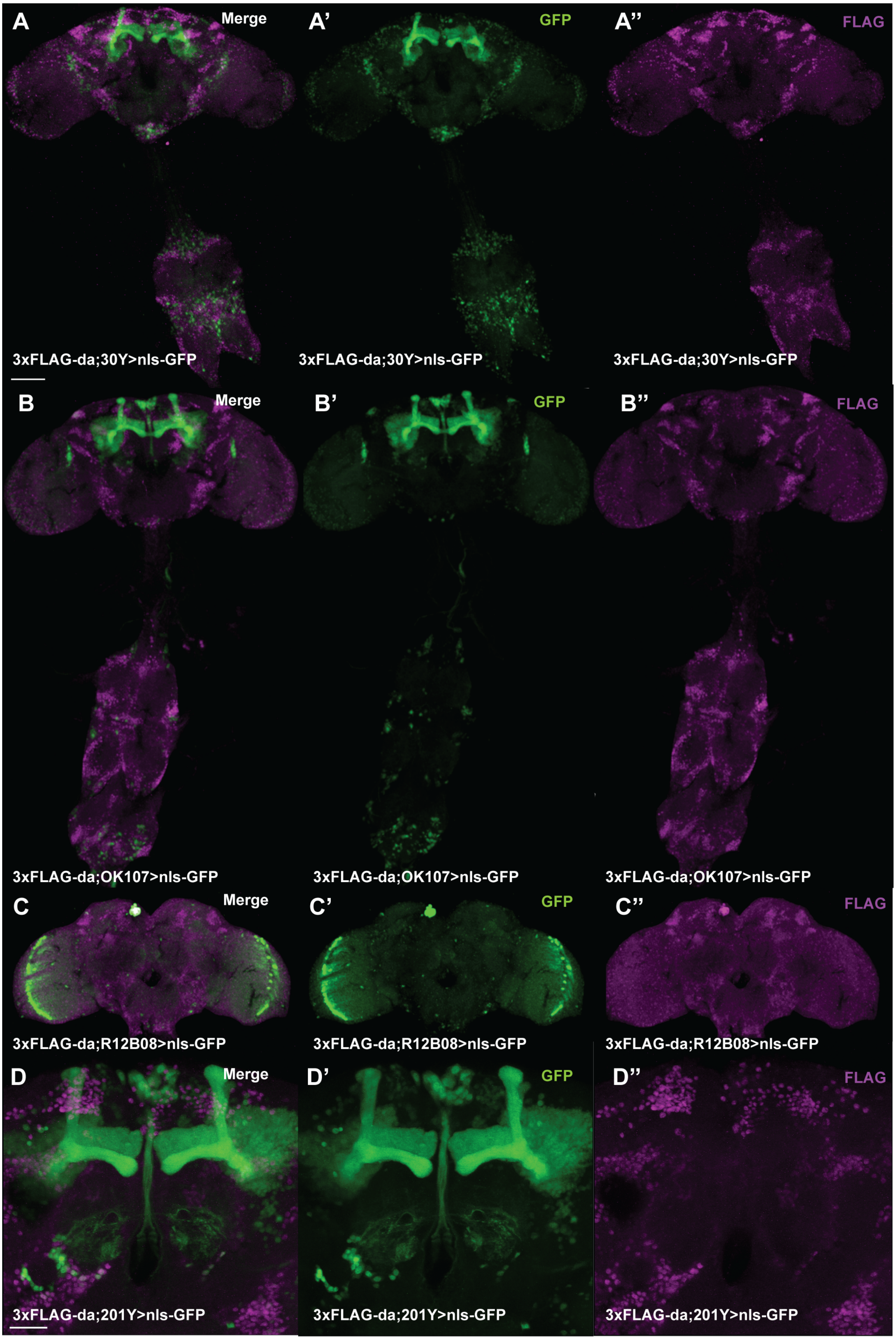

Disease Models & Mechanisms • Supplementary information

Figure S6. Da is expressed widely in the adult *Drosophila* brain including the central brain and thoracic ganglion. A-A'' – Da is expressed in many cells marked by *30Y-Gal4*. B-B'' Da is expressed in some cells marked by *OK107-Gal4*. C-C'' – Da is expressed in a few cells marked by *R12B08-Gal4.* D-D'' – Da is expressed in a few cells marked by *201Y-Gal4.* Driver expression is visualized with nls-GFP (A', B', C' and D') and 3xFLAG-Da is magenta (A'', B'', C'' and D''). Scale bar represents 70 μm on A and 30 μm on D.

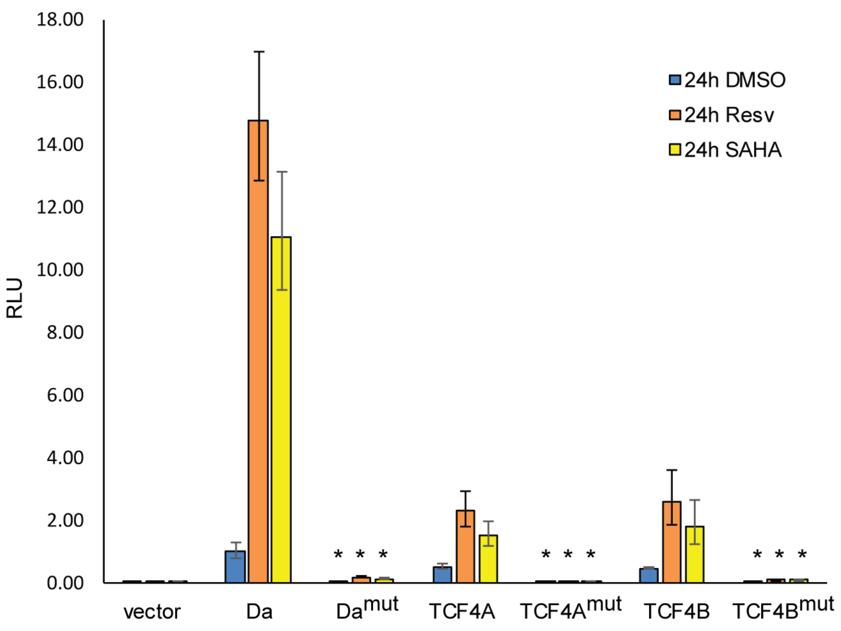

Figure S7. Mutations in bHLH domain of Daughterless and TCF4 abolish their transactivational capability. Cultured rat primary neurons were co-transfected with constructs encoding empty vector, wild type Da, R580W mutated Da (Da^{mut}), *TCF4A,* A587P mutated *TCF4A* ($TCF4A^{mut}$)*, TCF4B* or A587P mutated *TCF4B* ($TCF4B^{mut}$), firefly luciferase construct carrying 12 μE5 boxes with a *TK* promoter, and *Renilla* luciferase construct with mouse *PGK* promoter for normalization. Luciferase activities were measured and data are presented relative to the luciferase signals obtained from cells transfected with wild type *da*-encoding construct and treated with 0.1% DMSO for control. Mean results from three independent transfection experiments performed in duplicates are shown. Error bars show standard error of the mean (s.e.m). Statistical significance is shown compared to cells expressing respective non-mutated effector protein and treated similarly. *P<0.05, One Way ANOVA with post-hoc Bonferroni test.; RLU - relative luciferase unit.

Disease Models & Mechanisms • Supplementary information

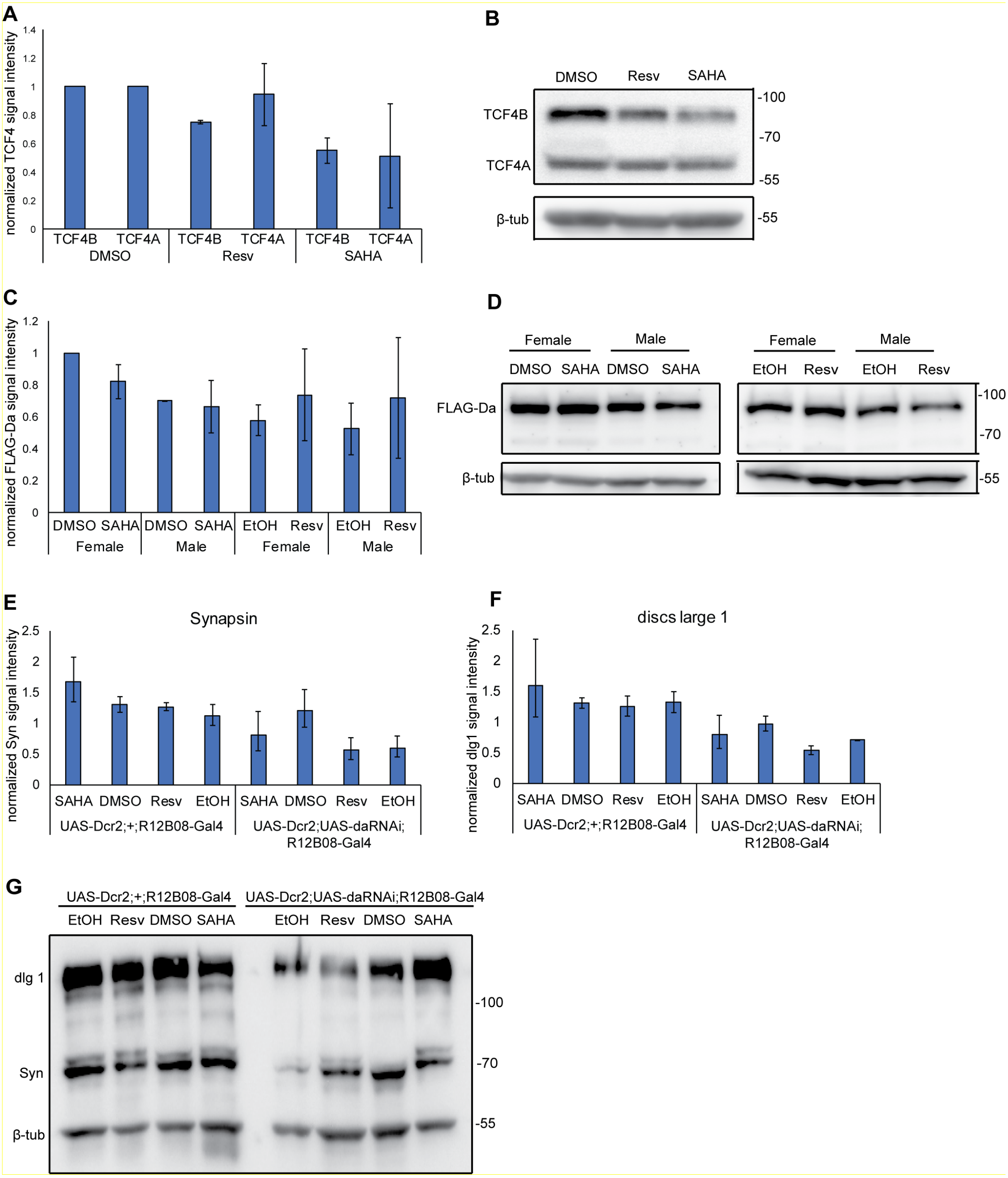

Figure S8. Resveratrol and SAHA do not increase levels of Daughterless, TCF4 and Daughterless target genes Synapsin and discs large 1. A – rat cortical neurons were treated with 50 µM resveratrol, 5 µM SAHA or 0.1% DMSO as control for 24h. Cells were lysed in 2x SDS sample buffer and equal amounts of protein extract was loaded to gel. For visualizing TCF4 ITF-2 Antibody (C-8) from Santa Cruz was used and the signals were normalized using β-tubulin signals, n=2. B – representative Western blot from rat cortical neurons. C – 0-24 h old *3xFLAG-da* flies were fed with 400 µM resveratrol or 2 µM SAHA, or 1% ethanol or 0.1% DMSO as control for 5 days. 5 fly heads were lysed in 2x SDS sample

Disease Models & Mechanisms • Supplementary information

buffer and loaded to gel. FLAG-Da signals were normalised using β-tubulin signals, n=2. D - representative Western blot from adult fly heads. E and F – larvae were grown in food substrate containing 400 μM resveratrol or 2 μM SAHA, or 1% ethanol or 0.1% DMSO as control. 20 3rd instar larval brains were lysed in 2x SDS sample buffer and loaded to gel. Synapsin and discs large 1 signals were normalized using β-tubulin signals, n=3. G – representative Western blot from Representative Western blot using 3rd instar larval brains. B, D and G - numbers indicate molecular weight of proteins in kDa.

Disease Models & Mechanisms • Supplementary information

Table S1. Used qPCR primers

Primers	***Forward***	***Reverse***
**Ac (ChIP)** (Andrade-Zapata and Baonza, 2014)	ggtatcagggcctagggatcc	gatccttcagtgatgatgctgttg
**PHM (ChIP)**	cagaccgtaagtccaggttcc	cgggaaaaaccagagcctat
**SynI (ChIP)**	ttcgagtggaaatttgtgagc	gccgtgtttcgatttttggg
**SynII (ChIP)**	ccggctaatgacaccaggaa	ggattggcaggtagacgagg
**Dlg1I (ChIP)**	gctgcaattgcgaagctaca	tggttcgcaaggtgcgatag
**Dlg1II (ChIP)**	cgtcgccagatacacgagtt	cgtggtccagcttggtactc
**Dlg1III (ChIP)**	tcactgtattcggttcttgcct	ccagtctgtgtgagttggct
**Dlg1IV (ChIP)**	gctgggcatctgcgttctat	acttggctagtgatcctgctc
**DaQ**	ggtggctcaacgtcaacact	atcgtcactggtcgccattt
**AcQ**	aagcaaggagcatcgtcaca	agagtgattttcgctgccca
**PHMQ**	cgatctgtacttgtgcacgc	tatggtgggccgtgttcatc
**SynQ**	gcgagggtctgaacaatcca	gtggtcttgctgtctccgaa
**Dlg1Q1**	ccaagttgatggacggcgga	aggttcttctcgctcccgtt
**Dlg1Q2**	gcttgtttcaagcgctgtt	atttgcaaggtctccgctgt
